# Foxp3^+^ Regulatory T Cells Delay Expulsion of Intestinal Nematodes by Suppression of IL-9-Driven Mast Cell Activation in BALB/c but Not in C57BL/6 Mice

**DOI:** 10.1371/journal.ppat.1003913

**Published:** 2014-02-06

**Authors:** Birte Blankenhaus, Martina Reitz, Yannick Brenz, Marie-Luise Eschbach, Wiebke Hartmann, Irma Haben, Tim Sparwasser, Jochen Huehn, Anja Kühl, Thorsten B. Feyerabend, Hans-Reimer Rodewald, Minka Breloer

**Affiliations:** 1 Bernhard Nocht Institute for Tropical Medicine, Hamburg, Germany; 2 Institute of Infection Immunology, TWINCORE, Centre for Experimental and Clinical Infection Research; a joint venture between the Helmholtz Centre for Infection Research Braunschweig and the Hanover Medical School, Hanover, Germany; 3 Experimental Immunology, Helmholtz Centre for Infection Research, Braunschweig, Germany; 4 Medizinische Klinik für Gastroenterologie, Infektiologie und Rheumatologie, Charité-Campus Benjamin Franklin, Berlin, Germany; 5 Division for Cellular Immunology, Deutsches Krebsforschungszentrum, Heidelberg, Germany; University of Edinburgh, United Kingdom

## Abstract

Accumulating evidence suggests that IL-9-mediated immunity plays a fundamental role in control of intestinal nematode infection. Here we report a different impact of Foxp3^+^ regulatory T cells (Treg) in nematode-induced evasion of IL-9-mediated immunity in BALB/c and C57BL/6 mice. Infection with *Strongyloides ratti* induced Treg expansion with similar kinetics and phenotype in both strains. Strikingly, Treg depletion reduced parasite burden selectively in BALB/c but not in C57BL/6 mice. Treg function was apparent in both strains as Treg depletion increased nematode-specific humoral and cellular Th2 response in BALB/c and C57BL/6 mice to the same extent. Improved resistance in Treg-depleted BALB/c mice was accompanied by increased production of IL-9 and accelerated degranulation of mast cells. In contrast, IL-9 production was not significantly elevated and kinetics of mast cell degranulation were unaffected by Treg depletion in C57BL/6 mice. By in vivo neutralization, we demonstrate that increased IL-9 production during the first days of infection caused accelerated mast cell degranulation and rapid expulsion of *S. ratti* adults from the small intestine of Treg-depleted BALB/c mice. In genetically mast cell-deficient (Cpa3-Cre) BALB/c mice, Treg depletion still resulted in increased IL-9 production but resistance to *S. ratti* infection was lost, suggesting that IL-9-driven mast cell activation mediated accelerated expulsion of *S. ratti* in Treg-depleted BALB/c mice. This IL-9-driven mast cell degranulation is a central mechanism of *S. ratti* expulsion in both, BALB/c and C57BL/6 mice, because IL-9 injection reduced and IL-9 neutralization increased parasite burden in the presence of Treg in both strains. Therefore our results suggest that Foxp3^+^ Treg suppress sufficient IL-9 production for subsequent mast cell degranulation during *S. ratti* infection in a non-redundant manner in BALB/c mice, whereas additional regulatory pathways are functional in Treg-depleted C57BL/6 mice.

## Author Summary

Parasitic worms are large multicellular organisms that manage completion of their life cycles despite exposure to their host's immune system. To avoid expulsion, parasitic worms actively suppress their host's immune response. Here we show that the pathogenic nematode *Strongyloides ratti* induces the expansion of a specialized subset of regulatory immune cells, regulatory T cells (Treg), that counteract effector T cell function. Treg expanded with similar kinetics and suppressed most features of the nematode-specific immune response in two different mouse strains, BALB/c and C57BL/6. One central factor of this immune response i.e. IL-9-driven rapid degranulation of mast cells, was suppressed by parasite-induced Treg in BALB/c mice non-redundantly. Consequently, Treg depletion elevated IL-9 production, accelerated mast cell degranulation and led to rapid expulsion of *S. ratti* in BALB/c mice. *S. ratti*-infected C57BL/6 mice still displayed low IL-9 production and delayed mast cell degranulation in the absence of Treg. Thus *S. ratti* was able to complete its life cycle in Treg-depleted C57BL/6 mice. This study shows that parasitic worms delay their expulsion by over-activating regulatory elements of their host's immune system such as Treg. The importance of individual regulatory elements during immune evasion depends on their degree of redundancy within the host that is variable in different genetic backgrounds.

## Introduction

Helminths are large multicellular parasites that may survive for years within their mammalian hosts despite their potential exposure to the immune system. This is achieved by active suppression of their host's immune response utilizing regulatory pathways that are intrinsic parts of the mammalian immune system [1], [2]. Thereby, helminths have been shown to secrete analogs of regulatory cytokines, to induce regulatory receptors on their host's leukocytes and to mediate expansion and activation of regulatory cell populations. Among these, regulatory T cells are the most prominent mediators of immunological homeostasis [3], [4]. Consequently, Treg numbers and suppressive capacity are increased in helminth-infected humans [5], [6], [7], [8], [9] and mice [10], [11], [12], [13], [14], [15]. Depletion or functional inactivation of Treg resulted in increased immune pathology [16], [17], [18], [19], reduced parasite burden [13], [14], and abrogated suppression of immune response to unrelated antigens in several murine helminth infection models [20], [21], [22], [23], thus suggesting that Treg may promote parasite survival by active immune suppression [24].

Treg can be identified by constitutive expression of the IL-2 receptor alpha chain (CD25) and more precisely by expression of the transcription factor forkhead box p3 (Foxp3) [25]. Treg depletion or inactivation by injection of mAb specific for CD25 may interfere with effector T cell function. To circumvent this problem mouse strains that express the human diphtheria toxin receptor (DTR) in Treg have been generated [26], [27]. Depletion of regulatory T cell (DEREG) mice are transgenic for a bacterial artificial chromosome that drives expression of a fusion protein consisting of the DTR and enhanced green fluorescent protein (eGFP) under the control of the Foxp3 promoter. Thus Foxp3^+^ Treg can be monitored by the eGFP expression in these mice and application of DT results in their rapid but transient depletion. Although this model is not feasible for long term or repeated Treg depletion, it allows the specific eradication of Foxp3^+^ Treg during a short period while activated effector T cells are not affected directly [28].

We use experimental infection of mice with the pathogenic nematode *Strongyloides ratti* to investigate the role of Foxp3^+^ Treg during helminth infection. This rodent parasite is a suitable model for geohelminth infections, displaying tissue migrating and intestinal life stages [29]. *S. ratti* infective third stage larvae (iL3) penetrate the skin of their rodent host and migrate within 2 days to the head. iL3 are swallowed, reach the intestine and molt via a fourth larval stage to parasitic adults by day 5 post infection (p.i.). Parasites live embedded in the mucosa of the small intestine and reproduce by parthenogenesis. Eggs and already hatched first stage larvae (L1) leave the host with the feces by day 5–6 p.i. Female L1 may directly develop into iL3 and invade another host or molt to free living adults that reproduce sexually for one generation. Immune competent mice and rats resolve the infection spontaneously within 2–4 weeks and remain semi-resistant to subsequent infections [30], [31]. Experimental infection of mice and rats induces a protective type 2 immune response characterized by induction of the cytokines IL-4, IL-5 and IL-13 as well as production of nematode-specific IgM and IgG1 [32], [33]. Migrating larvae in the tissue are opsonized by antibodies and complement and eradicated by granulocytes [34], [35], [36], [37]. Final expulsion of parasitic adults from the small intestine is thought to be promoted by mast cells [38], [39], [40] although a conclusive demonstration of protective roles of mast cells would require the use of Kit-independent models [41]. Moreover, the role of IL-9-mediated immunity in eradication of migrating larvae or parasitic adults has not been investigated so far.

To date DEREG mice have been used to analyze the role of Foxp3^+^ Treg during infection of mice with two different nematodes, *S. ratti* and *Heligmosomoides polygyrus*, an orally transmitted gastrointestinal parasite [14], [18]. Both, *S. ratti* and *H. polygyrus* infection increased Treg numbers but did not change the frequency of Treg within the CD4^+^ T cell compartment as effector T cells expanded with similar kinetics. Transient Treg depletion either in C57BL/6 DEREG mice during days 4 to 6 of *H. polygyrus* infection or in BALB/c DEREG mice during days 0 to 2 of *S. ratti* infection increased the nematode-specific cellular immune response. Improved immune response did not reduce *H. polygyrus* parasite burden analyzed day 14 p.i. but aggravated pathology in the small intestine [18]. In contrast, improved immune response to *S. ratti* was correlated with dramatically reduced parasite burden in the small intestine and equally reduced larval output in the feces throughout infection [14]. Treg depletion did not improve eradication of tissue migrating *S. ratti* larvae but induced rapid expulsion of parasitic adults in the context of accelerated mast cell degranulation. Despite the different final outcome, both studies highlight the general importance of nematode-induced Treg in suppressing the host's immune response to prevent either expulsion and/or induction of immune pathology.

The different effect of Treg depletion on resistance may be explained by the different infection models and variations in timing and methodology of Treg depletion [24]. A possible impact of the genetic background of the murine host, however, has not been investigated systematically so far.

Here, we compare the effect of Treg depletion during *S. ratti* infection in BALB/c DEREG and C57BL/6 DEREG mice. Although Treg expanded with comparable kinetics, displayed a comparable phenotype and equally suppressed the nematode-specific humoral and cellular type 2 immune response parasite burdens were selectively reduced in BALB/c but not in C57BL/6 mice upon Treg depletion. We show that improved resistance in Treg-depleted BALB/c mice was mediated by increased IL-9 production and the protection required mast cells. In line with recent data suggesting that IL-9 plays a fundamental role in control of helminth infection and pathology [42], [43], we confirm that IL-9 is central for control of *S. ratti* infection in both, BALB/c and C57BL/6 mice. We demonstrate that Foxp3^+^ Treg control this axis of IL-9 production leading to mast cell-driven parasite expulsion in BALB/c mice. As IL-9 production was not significantly elevated upon Treg depletion in C57BL/6 mice, we further demonstrate genetically determined differences in control of endogenous IL-9 production.

## Results

### *S. ratti* infection induces transient expansion of Foxp3^+^ Treg in BALB/c and C57BL/6 mice

**Figure 1:**
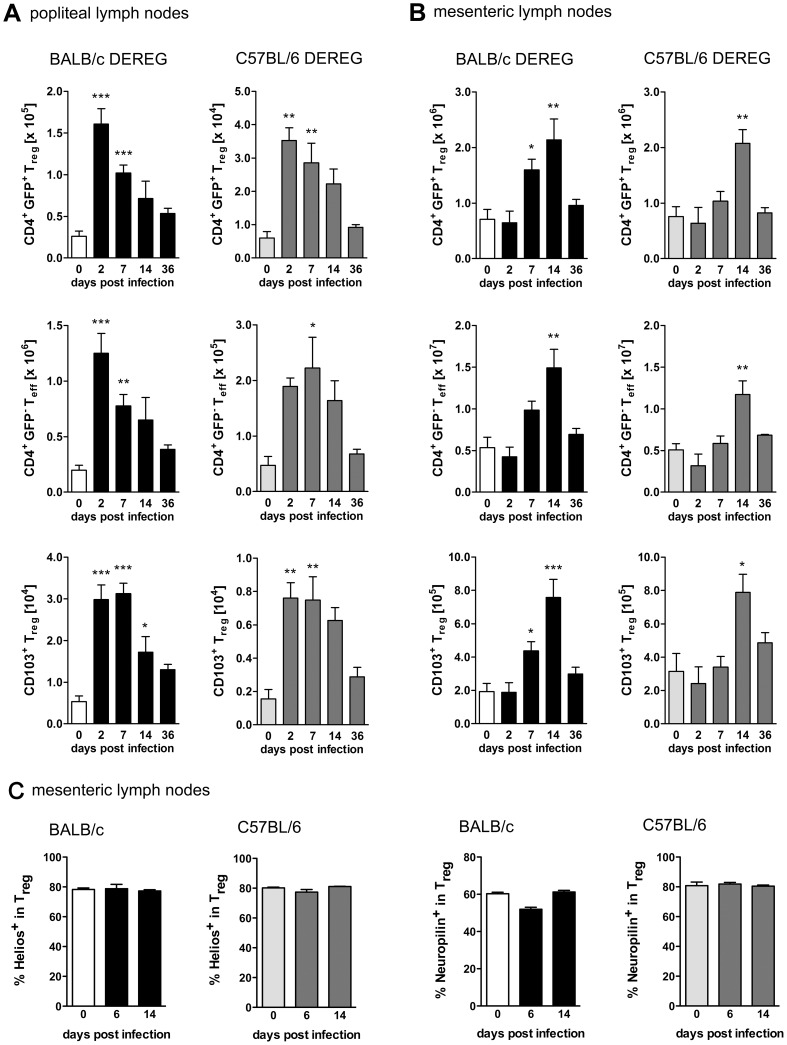
Treg expansion during *S. ratti* infection. BALB/c DEREG and C57BL/6 DEREG mice were infected s.c. with 2000 *S. ratti* iL3. Mice were sacrificed at the indicated time points. Lymph node cells were stained for CD4 and CD103 (**AB**) or CD4, Foxp3, Helios and Neuropilin (**C**). Gating strategy is shown in Figure S1. **AB:** Graphs show numbers of Treg (upper panel, CD4^+^GFP^+^), Teff (middle panel, CD4^+^GFP^−^) and activated Treg (lower panel CD103^+^CD4^+^GFP^+^) in popliteal (**A**) and mesenteric (**B**) lymph nodes at indicated time points of infection in BALB/c DEREG (left) and C57BL/6 DEREG (right) mice. **C:** Graphs show frequency of Helios^+^ and Neuropilin^+^ cells in the CD4^+^Foxp3^+^ Treg population in mesenteric lymph nodes of BALB/c and C57BL/6 mice at the indicated time points of infection. Shown are the combined results of two independent experiments (**AB:** n = 7 and **C:** n = 4–6). Asterisks indicate significant difference of the mean of infected mice compared to naïve mice (day 0 p.i.) analyzed by one-way ANOVA with Bonferroni post test (* p≤0.05, ** p≤0.01, *** p≤0.001).

To compare the role of Foxp3^+^ Treg in different mouse strains, BALB/c DEREG and C57BL/6 DEREG mice were infected by s.c. injection of *S. ratti* iL3 in the footpad. Foxp3^+^ Treg were identified as CD4^+^GFP^+^ lymphocytes at different time points during infection (Figure S1A). *S. ratti* infection led to a rapid but transient increase in Treg numbers, first in the popliteal lymph nodes (PLN) that drain the site of infection (Figure 1A) and later in the mesenteric lymph nodes (MLN) that drain the small intestine where adult parasites are located (Figure 1B). Upon direct comparison to C57BL/6 mice, BALB/c mice displayed an earlier Treg expansion in the MLN but both strains showed maximal Treg numbers by day 14 p.i. Expanding Treg displayed an activated effector-memory phenotype in both strains indicated by expression of the integrin CD103 [44] (Figure 1A, B and Figure S1B). Treg numbers returned to naïve levels once infection was resolved i.e. by day 36 p.i. As the numbers of CD4^+^GFP^−^ effector T cells expanded and contracted with similar kinetics (Figure 1A and 1B), the frequencies of Treg within the CD4^+^ T cell compartment remained constant in PLN and MLN in both mouse strains (data not shown). Expression of Helios and Neuropilin (CD304) has been used to distinguish thymus-derived Treg from Treg that were induced in the periphery [45], [46], [47]. The frequency of Helios^+^ and Neuropilin^+^ Treg in MLN (Figure 1C and S1C) and spleen (data not shown) of BALB/c and C57BL/6 mice did not change during *S. ratti* infection thus suggesting no differences in the origin of expanding Treg in both strains.

### Treg depletion improves resistance to *S. ratti* infection selectively in BALB/c mice

**Figure 2:**
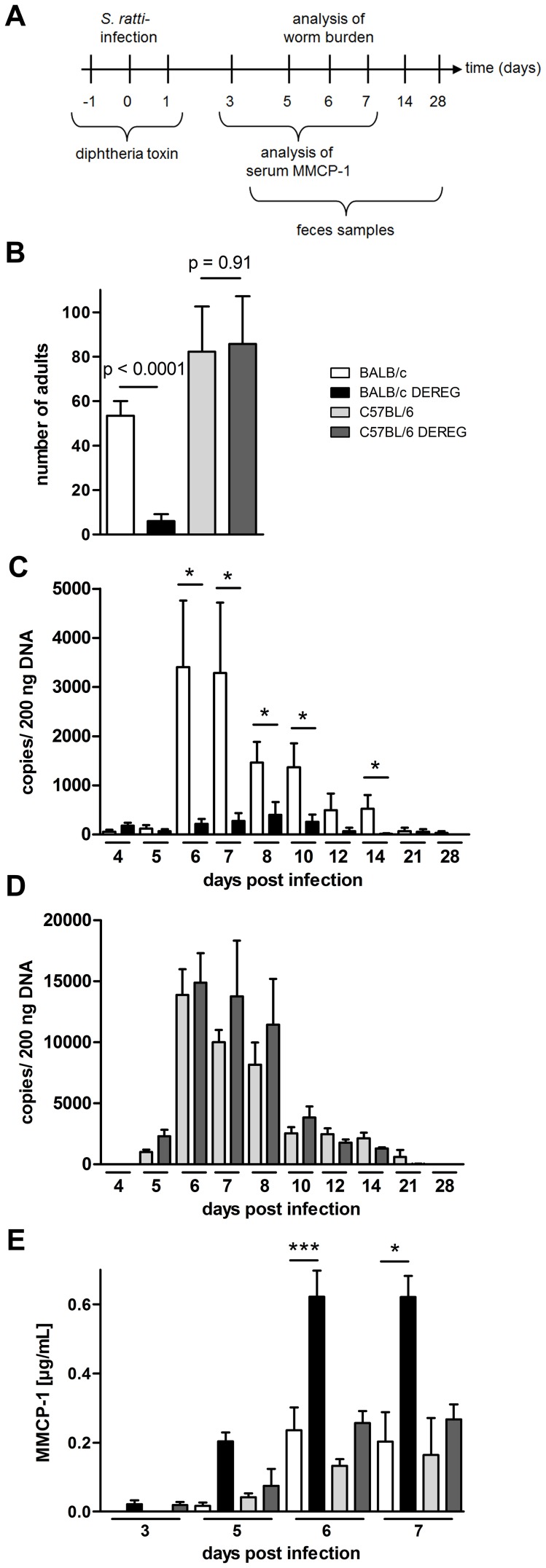
Increased resistance to S. *ratti* infection in Treg-depleted BALB/c DEREG but not in C57BL/6 DEREG mice. BALB/c (white bars), BALB/c DEREG (black bars), C57BL/6 (light grey bars), and C57BL/6 DEREG (dark grey bars) mice were treated with DT and infected s.c. with 2000 *S. ratti* iL3. **A:** Experimental setup is shown. **B:** Number of parasitic adults in the small intestine was counted on day 6 p.i. Shown are the combined results of two independent experiments (n = 10). Numbers show difference of the mean analyzed by students *t* test. **CD:** release of *S. ratti*-derived DNA in the feces over 24 h was quantified at the indicated time points in DT treated BALB/c and BALB/c DEREG (**C**) or C57BL/6 and C57BL/6 DEREG (**D**). Shown are the means of 5 mice per time point and group. This result is representative for two independent experiments. Asterisks show significant difference of the mean analyzed by students *t* test (* p≤0.05). **E:** MMCP-1 in the serum of infected mice was quantified at the indicated time points. Shown are the combined results of three independent experiments (n = 10 for day 7; n = 9 for days 6 and 3; n = 8 for day 5). Asterisks indicate significant difference of the mean analyzed by one-way ANOVA with Bonferroni post test (* p≤0.05, *** p≤0.001).

Transient depletion of Foxp3^+^ Treg during the first days of *S. ratti* infection was achieved by three consecutive injections of DT in DEREG mice or non-transgenic littermates (Figure 2A). Consistent with our previous results [14], Treg depletion in BALB/c DEREG mice resulted in reduced parasite burden in the small intestine at day 6 p.i. Strikingly, Treg depletion within the same infection experiments did not change parasite burden in C57BL/6 DEREG mice (Figure 2B). To monitor the course of *S. ratti* infection, we quantified the released eggs and first stage larvae in the feces by qPCR specific for the *Strongyloides* 28S RNA gene [33]. Reduced numbers of parasitic adults in the small intestine of Treg-depleted BALB/c DEREG mice were reflected by reduced larval output throughout infection as we had shown before [14] (Figure 2C). We did not record statistically significant differences in the larval output of C57BL/6 mice in the presence or absence of Treg until clearance of infection at day 28 p.i. (Figure 2D). Thus Treg depletion in C57BL/6 mice did not improve host defense at any time point of infection.

As C57BL/6 mice were more susceptible to *S. ratti* infection, we compared the outcome of Treg depletion in C57BL/6 mice that received a lower infection dose to BALB/c mice that received the standard infection dose. Treg depletion did not reduce parasite burden in C57BL/6 mice carrying less than 10 parasitic adults in the small intestine while a mean parasite burden of 20 adults in BALB/c mice was reproducibly reduced by Treg depletion (Figure S2).

Rapid clearance of *S. ratti* in Treg-depleted BALB/c DEREG mice is not achieved by improved killing of migrating larvae in the tissue but by enhanced expulsion of adult parasites from the small intestine [14]. Studies using Kit-mutant mice suggested that efficient expulsion of *S. ratti* and *S. venezuelensis* adults from the small intestine depends on mast cells [38], [48], and improved parasite expulsion in Treg-depleted BALB/c mice was correlated to increased and accelerated mast cell degranulation [14]. Therefore, we recorded the concentration of mouse mast cell protease-1 (MMCP-1) that is released upon degranulation of mucosal mast cells [49] in the serum of BALB/c and C57BL/6 mice (Figure 2E). Both mouse strains displayed comparable increases in circulating MMPC-1 in the presence of normal Treg frequencies. Depletion of Foxp3^+^ Treg in BALB/c DEREG mice reproducibly increased and accelerated this mast cell degranulation, yielding maximal MMCP-1 concentrations in the serum by day 6 p.i. In sharp contrast, Treg depletion had no impact on mast cell degranulation in C57BL/6 DEREG mice.

**Figure 3:**
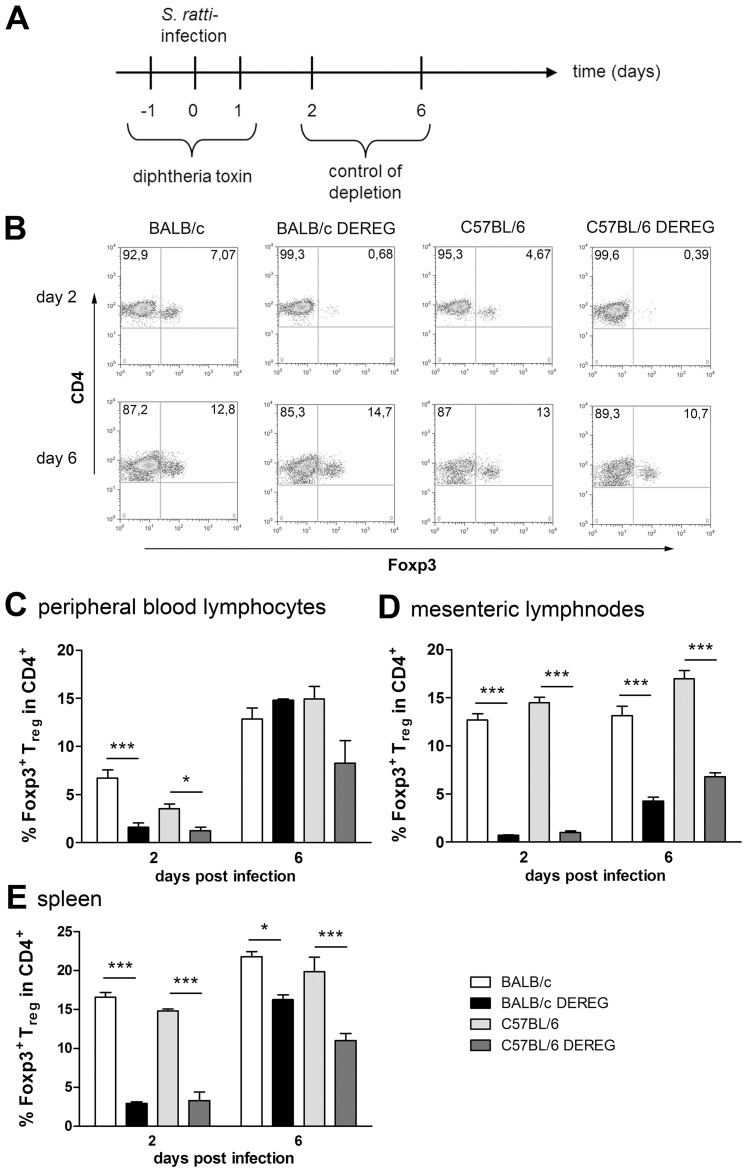
Depletion efficacy of Treg in BALB/c DEREG and C57BL/6 DEREG mice. BALB/c (white bars), BALB/c DEREG (black bars), C57BL/6 (light grey bars), and C57BL/6 DEREG (dark grey bars) mice were treated with DT and infected s.c. with 2000 *S. ratti* iL3. Mice were sacrificed days 2 and 6 p.i. and peripheral blood lymphocytes, mesenteric lymph node cells and spleen cells were stained for Foxp3 and CD4. **A:** Experimental setup is shown. **B:** Shown are representative dotplots of CD4/Foxp3 staining on day 2 p.i. (upper panel) and day 6 p.i. (lower panel) in peripheral blood lymphocytes. **C–E:** Percentage of Foxp3^+^ cells in CD4^+^ T cells at indicated time points in PBL (**C**) mesenteric lymph nodes (**D**) and spleen (**E**). The experiment shown (n = 4) is representative for 3 repeats; Asterisks indicate significant difference of the mean analyzed by one-way ANOVA with Bonferroni post test (* p≤0.05, *** p≤0.001).

The absent effect of Treg depletion in C57BL/6 DEREG mice was not due to differences in depletion efficacy or repopulation kinetics (Figure 3). Our transient depletion regime (Figures 3A) reduced the frequency of Foxp3^+^ Treg within the CD4^+^ T cell population by 90% until day 2 p.i. in both strains (Figure 3B–E). Repopulation of the Treg compartment occurred rapidly in peripheral blood (Figure 3C) and spleen (Figure 3E) and with slower kinetics in the MLN (Figure 3D). Comparison of Foxp3^+^ Treg repopulation velocity in BALB/c and C57BL/6 mice revealed no differences (Figures 3C–E). The missing effect of Treg depletion in C57BL/6 DEREG mice also does not reflect different susceptibility of BALB/c and C57BL/6 mice to DT-mediated side effects, since DT treatment had no effect in non-transgenic littermates of both strains. Taken together these results show that despite comparable induction of Foxp3^+^ Treg numbers during *S. ratti* infection, Treg depletion was translated into accelerated mast cell degranulation and improved expulsion of parasitic adults selectively in BALB/c mice.

### Improved resistance in Treg-depleted BALB/c mice is not mediated by granulocytes

**Figure 4:**
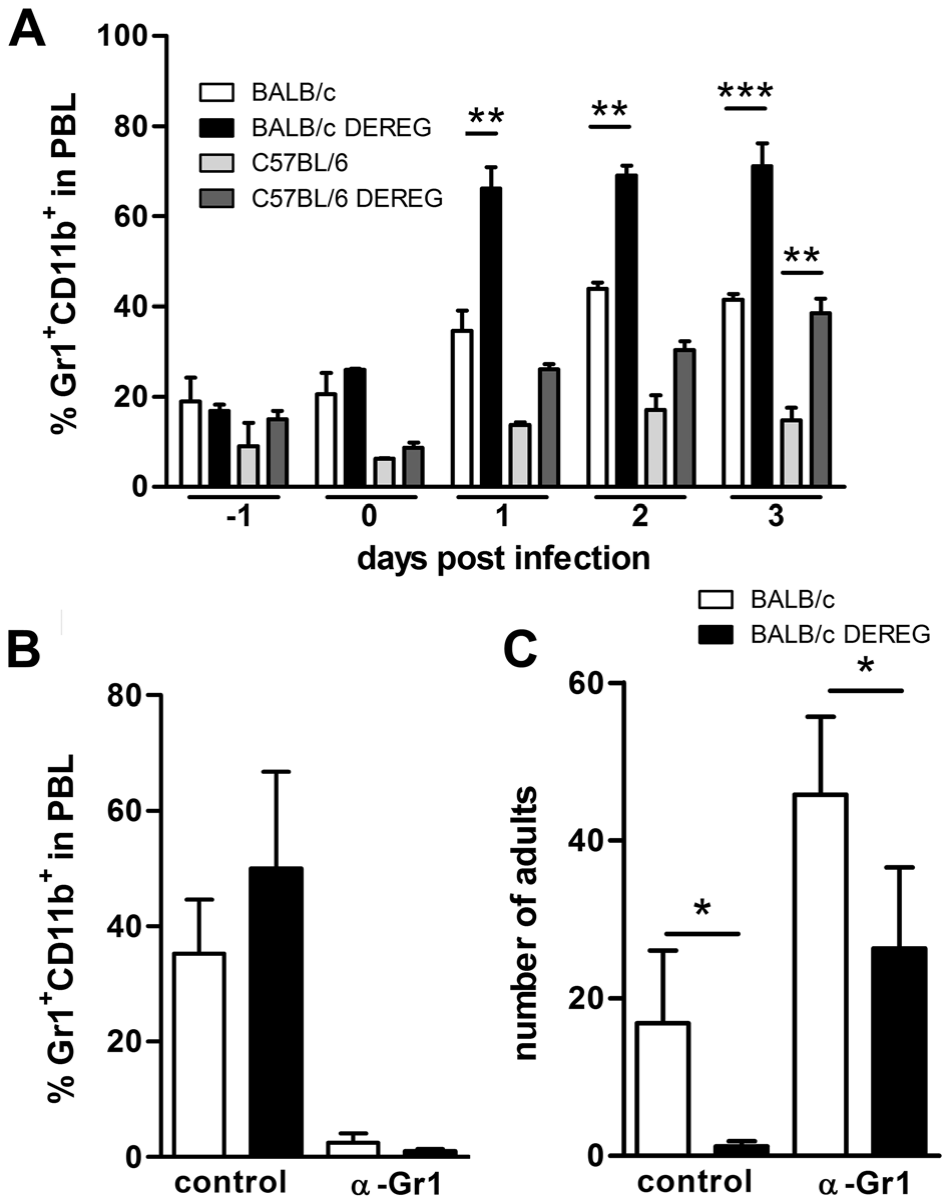
Improved resistance in Treg-depleted BALB/c DEREG mice in the absence of granulocytes. BALB/c (white bars), BALB/c DEREG (black bars), C57BL/6 (light grey bars), and C57BL/6 DEREG (dark grey bars) mice were treated with DT on 3 consecutive days starting one day before *S. ratti* infection. **A:** Percentage of Gr1^+^CD11b^+^ cells in PBL was analyzed at the indicated time points. **BC:** BALB/c (white bars), BALB/c DEREG (black bars) were treated with DT and received an additional injection of anti-Gr1 mAb or isotype control one day before *S. ratti* infection. Percentage of Gr1^+^CD11b^+^ cells in PBL of infected non-depleted mice was measured on day 1 p.i. (**B**) and number of adult parasitic females in the small intestine was counted on day 6 p.i. (**C**). Shown are the combined results of two independent experiments (n = 6). Asterisks indicate significant difference of the mean analyzed by students *t* test (* p≤0.05).

We recognize that depletion of 2–5% of leukocytes in vivo may cause effects that are independent of nature and function of the depleted cell type. In this context it was shown that DT-mediated depletion of dendritic cells caused neutrophilia as a side effect [50]. Increased number of neutrophils subsequently improved control of the employed ultra pathogenic *Escherichia coli* infection model independent of presence or absence of dendritic cells. We observed a two-fold increase in the frequency of Gr1^+^CD11b^+^ granulocytes in the peripheral blood of both DT treated BALB/c DEREG and C57BL/6 DEREG mice (Figure 4A). As granulocytes have been shown to kill migrating *S. ratti* larvae in the tissue [34] and the absolute expansion of granulocytes in the peripheral blood was more pronounced in BALB/c mice compared to C57BL/6 DEREG mice, we wanted to test if the differential mobilization of granulocytes caused the improved resistance in Treg-depleted BALB/c DEREG mice. To this end we depleted Gr1^+^ cells by anti-Gr1 mAb injection in DT treated BALB/c DEREG and non-transgenic littermates (Figure 4B) one day before infection. Depletion of granulocytes increased parasite burden in general, i.e. in the absence and presence of Treg (Figure 4C). This finding most likely reflects the higher number of larvae that reach the small intestine in the absence of attacking granulocytes. Additional Treg depletion in anti-Gr1 treated mice on the background of a higher *S. ratti* inoculum still resulted in a statistically significant decrease in the number of parasitic adults in the intestine (Figure 4C). Also depletion of Gr1^+^ cells after the tissue migrating phase, i.e. at day 3 p.i., did not abrogate the beneficial effect of Treg depletion in BALB/c mice (Figure S3). Although we cannot formally exclude a contribution of granulocytes to final expulsion of *S. ratti* from the small intestine in the presence and absence of Treg, these results show that improved resistance to *S. ratti* infection in Treg-depleted BALB/c DEREG mice did not depend on granulocytes and thus was not caused by additional mobilization of granulocytes in DT treated DEREG mice.

### Treg depletion improves *S. ratti*-specific B and T cell responses

**Figure 5:**
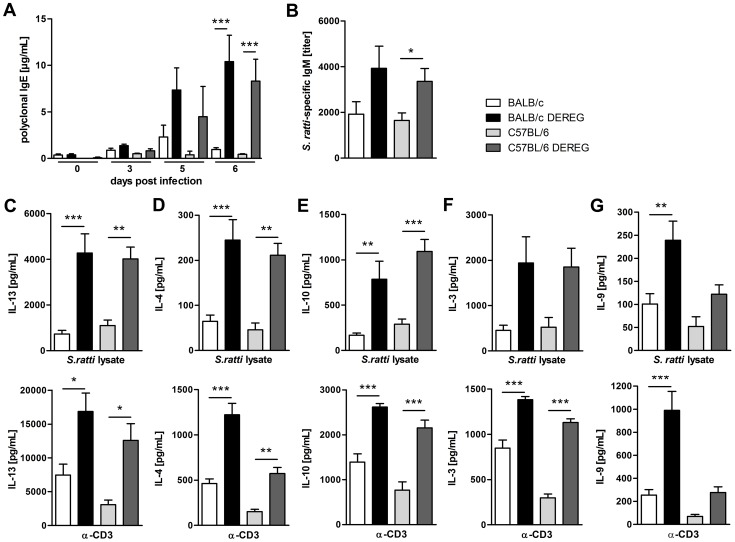
Immune response in the presence and absence of Treg during *S. ratti* infection. BALB/c (white bars), BALB/c DEREG (black bars), C57BL/6 (light grey bars), and C57BL/6 DEREG (dark grey bars) mice were treated with DT on three consecutive days starting one day before *S. ratti* infection. **A**: Polyclonal IgE in the serum was quantified at the indicated time points. Shown is one experiment (n = 6 per time point) that is representative for one independent repeat. Asterisks indicate significant difference of the mean analyzed by one-way ANOVA with Bonferroni post test (*** p≤0.001). **B:** *S. ratti*-specific IgM was quantified in the serum on day 7 p.i. Shown are the combined results of three independent experiments (n = 9–11). Asterisks indicate significant difference of the mean analyzed by students *t* test (* p≤0.05). **C–G:** Mice were sacrificed on day 6 p.i. and splenocytes (2×10^5^) were stimulated for 72 h with *S. ratti* lysate (upper panel) or with α-CD3 (lower panel). IL-13 (**C**), IL-4 (**D**), IL-10 (**E**), IL-3 (**F**) and IL-9 (**G**) in the supernatant were quantified by ELISA. Splenocytes that were cultivated in the presence of medium only did not secrete cytokines (data not shown). Shown are the combined results of two independent experiments (n = 8) for **C–F** and combined results of four independent experiments (n = 14–16) for **G**. Asterisks indicate significant difference of the mean analyzed by one-way ANOVA with Bonferroni post test (* p≤0.05, ** p≤0.01, *** p≤0.001).

Since control of *Strongyloides* infection was reported to depend on a Th2 immune response [51], [52], we compared the *S. ratti*-specific B and T cell response in BALB/c and C57BL/6 DEREG mice in the presence and absence of Treg. Concentrations of total IgE (Figure 5A) in the serum increased in the absence of Treg in both mouse strains. The early *S. ratti*-specific IgM response had slightly increased titers upon Treg depletion in BALB/c (p = 0.09) and C57BL/6 (p = 0.02) mice (Figure 5B). Antigen-specific IgG1 was not detectable at day 7 p.i. (data not shown). Antigen-specific stimulation of splenocytes derived from BALB/c and C57BL/6 mice that had been infected for 6 days induced a comparable production of the Th2 signature cytokines IL-13 and IL-4 (Figure 5C, 5D, upper panel). Treg depletion led to a clearly increased production of these cytokines in both mouse strains. Likewise, production of antigen-specific IL-10 increased upon Treg depletion in BALB/c and C57BL/6 DEREG mice to the same extent (Figure 5E, upper panel). Also IL-13, IL-4 and IL-10 responses to CD3 engagement i.e. polyclonal T cell activation increased in both strains in the absence of Foxp3^+^ Treg (Figure 5C–E, lower panel).

In order to understand the differential impact of Treg depletion on mast cell degranulation in BALB/c and C57BL/6 mice we analyzed the production of IL-3 and IL-9. IL-3 functions as growth factor for basophils and mast cells and was shown to contribute to expulsion of *Strongyloides venezuelensis* [53], [54]. Treg depletion increased IL-3 production in response to *S. ratti* antigen and to CD3 engagement in BALB/c and C57BL/6 mice to the same extent (Figure 5F), thus providing no explanation for the observed difference in mast cell activation.

IL-9 is a cytokine with pleiotropic function that also contributes to mastocytosis and mast cell activation [55]. BALB/c mice responded to Treg depletion with a statistically significant increase in IL-9 production in response to either antigen-specific stimulation or polyclonal T cell stimulation by CD3 engagement (Figure 5G). A weaker but statistically not significant up-regulation of IL-9 was also observed in Treg-depleted C57BL/6 DEREG mice. C57BL/6 mice produced generally less IL-9 than BALB/c mice, and the amount of IL-9 produced in the absence of Treg in C57BL/6 DEREG mice did not exceed the amount of IL-9 produced in BALB/c mice with normal Treg frequencies.

The different impact of in vivo Treg depletion on IL-9 secretion was not observed in vitro. Purified BALB/c- and C57BL/6-derived Treg suppressed IL-2 but also IL-9 secretion by splenocytes derived from *S. ratti*- mice in syngenic and allogenic combination to the same extent (Figure S4). Thus the differential regulation of IL-9 was, at least in vitro, not Treg-intrinsic but reflected either a limited capacity of C57BL/6 mice to produce IL-9 (Figure 5G) or the presence of redundant regulatory pathways in C57BL/6 mice that maintained control of IL-9 production in the absence of Foxp3^+^ Treg.

Taken together, depletion of Foxp3^+^ Treg in vivo increased most effectors of the adaptive type 2 immune response to *S. ratti* infection in BALB/c and C57BL/6 mice to the same extent. Among the parameters analyzed in this study, selectively IL-9 production and mast cell degranulation were significantly increased in Treg-depleted BALB/c mice. This leads to the question if the improved resistance in Treg-depleted BALB/c mice was caused by this increase in IL-9 production and the subsequent mast cell degranulation.

### IL-9 promotes *S. ratti* expulsion in BALB/c and C57BL/6 mice

**Figure 6:**
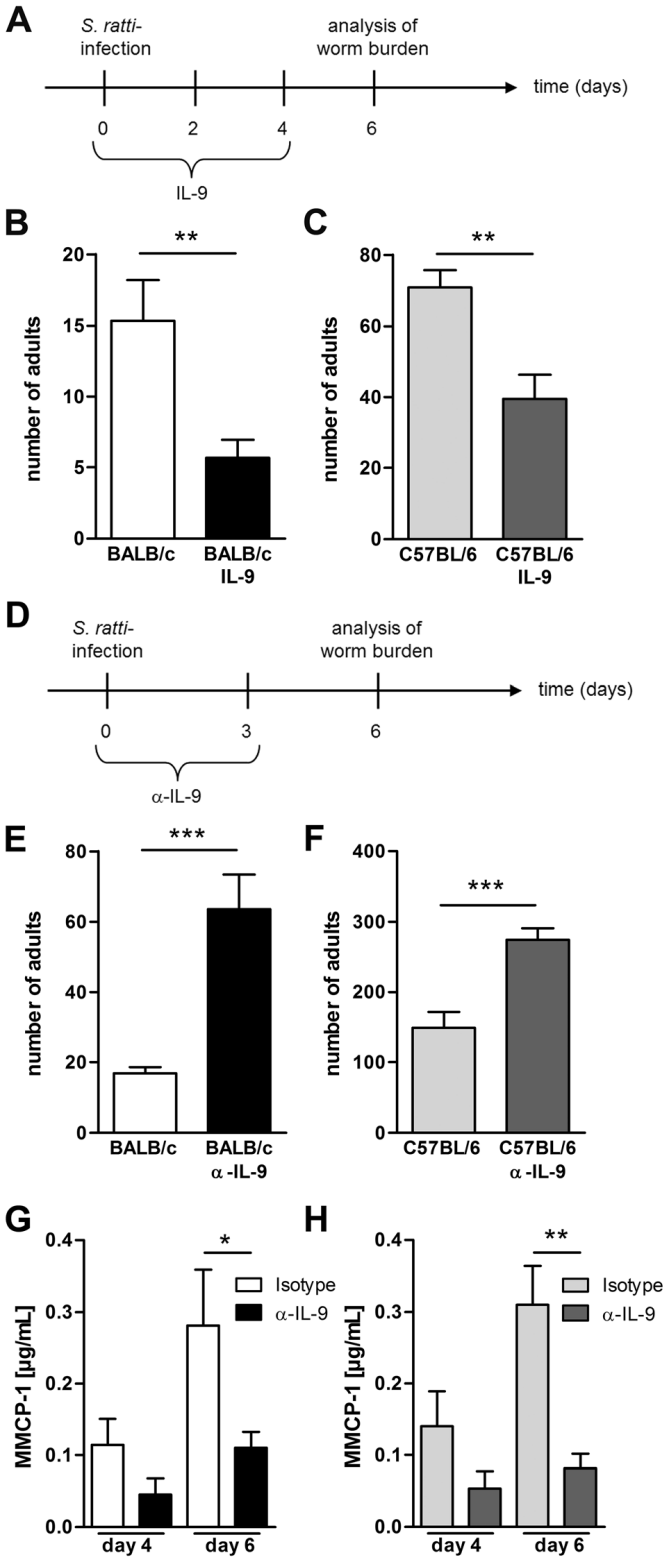
IL-9 administration or depletion during *S. ratti* infection. **A:** Experimental setup for IL-9 treatment is shown. **B–C:** Number of parasitic adults in the small intestine of infected non-treated BALB/c (**B**, white) and C57BL/6 (**C**, light grey) mice or infected and IL-9 treated BALB/c (**B**, black) and C57BL/6 (**C**, dark grey) mice on day 6 p.i. Shown are the combined results of three independent experiments (n = 12). **D:** Experimental setup for α-IL-9 treatment is shown. **E–F:** Number of parasitic adults in the small intestine of infected isotype treated BALB/c (**D**, white) and C57BL/6 (**E**, light grey) mice or infected and α-IL-9 treated BALB/c (**D**, black) and C57BL/6 (**E**, dark grey) mice on day 6 p.i. **G–H**: Concentration of MMCP-1 in the serum of infected isotype treated or α-IL-9 treated BALB/c (**G**) and C57BL/6 (**H**) mice on day 4 and day 6 p.i. Shown are the combined results of two independent experiments (n = 8). Asterisks indicate significant differences of the mean analyzed by students *t* test (** p≤0.01, *** p≤0.001).

A recent study demonstrated a fundamental role for IL-9 in control of *Nippostrongylus brasiliensis* infection in BALB/c mice [42]. However, the importance of IL-9 in control of gastrointestinal nematode infection depends on the nematode species and the genetic background of the host [56], [57], [58], [59], [60]. As the role of IL-9 in immunity to *S. ratti* infection has not been investigated so far, we first compared the impact of either recombinant IL-9 treatment or IL-9 neutralization in BALB/c and C57BL/6 mice (Figure 6). Forced increase of systemic IL-9 concentration by injection of IL-9 (Figure 6A) resulted in a significant reduction of parasitic adults in the small intestine of BALB/c (Figure 6B) and also C57BL/6 mice (Figure 6C). Neutralization of endogenous IL-9 that was produced during *S. ratti* infection resulted in a reciprocally increased parasite burden in both strains. (Figure 6D–F). IL-9 neutralization also reduced mucosal mast cell degranulation in BALB/c and C57BL/6 mice, demonstrating that endogenous IL-9 was required for degranulation (Figure 6G, 6H). These results show that IL-9-driven mast cell degranulation was associated with rapid expulsion of *S. ratti* in BALB/c and C57BL/6 mice. The fact that Treg depletion increased production of IL-9 in BALB/c DEREG mice but much less in C57BL/6 DEREG mice (Figure 5G) strongly suggests that this increased IL-9 contributed to improved resistance in the BALB/c strain.

### Foxp3^+^ Treg control early IL-9 production in BALB/c mice in vivo

**Figure 7:**
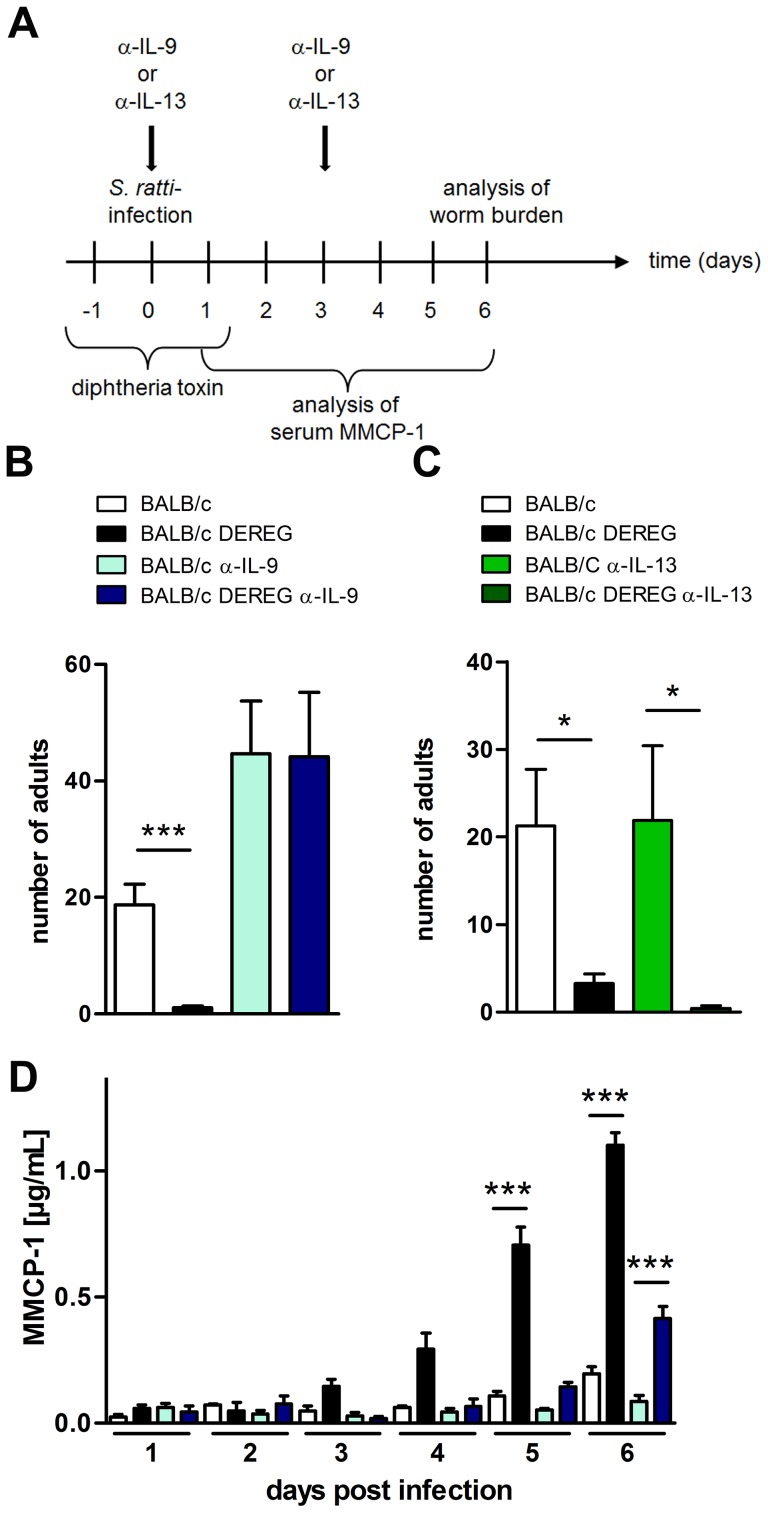
Role of IL-9 during *S. ratti* infection in Treg-depleted BALB/c mice. **A:** Experimental setup is shown. **B:** Number of parasitic adults in the small intestine of BALB/c (white), Treg-depleted BALB/c DEREG (black), α-IL-9 treated BALB/c (light blue) and α-IL-9 treated Treg-depleted BALB/c DEREG (dark blue) mice on day 6 p.i. **C:** Number of parasitic adults in the small intestine of BALB/c (white), Treg-depleted BALB/c DEREG (black), α-IL-13 treated BALB/c (light green) and α-IL-13 treated Treg-depleted BALB/c DEREG (dark green) mice on day 6 p.i. **D:** Concentration of MMCP-1 in the serum of infected mice at indicated time points. Shown are the combined results of four (**BD**) or two (**C**) independent experiments (BD: n = 14, C: n = 8). Asterisks indicate significant difference of the mean analyzed by (**BC**) students *t* test or (**D**) one-way ANOVA with Bonferroni post test (*** p≤0.001).

To test if enhanced resistance in Treg-depleted BALB/c mice was directly caused by the increased IL-9 production, we neutralized IL-9 and, as a control Th2 cytokine, IL-13 during Treg depletion in *S. ratti*-infected BALB/c DEREG mice (Figure 7A). As expected, Treg depletion reduced the parasite burden in the small intestine at day 6 p.i. (Figure 7B, 7C) and accelerated the degranulation of mast cells in the presence of endogenous IL-9 (Figure 7D). Additional neutralization of IL-9, but not neutralization of IL-13, abrogated this enhanced resistance. Numbers of parasitic *S. ratti* adults in the small intestine were alike in anti-IL-9 treated BALB/c mice in the presence and absence of Foxp3^+^ Treg (p = 0.97; Figure 7B) whereas Treg depletion still led to drastically reduced numbers of parasitic adults in anti-IL-13 treated mice (Figure 7C). Neutralization of endogenous IL-9 also abrogated the increased mast cell degranulation in Treg-depleted BALB/c mice until day 5 p.i.

One day later, at day 6 p.i., mast cell degranulation was also increased in Treg-depleted BALB/c mice that received anti-IL-9 treatment, although parasite burdens were not reduced in this group. This finding suggests that mast cell degranulation during the first days of infection was relevant for improved resistance in Treg-depleted BALB/c mice.

**Figure 8:**
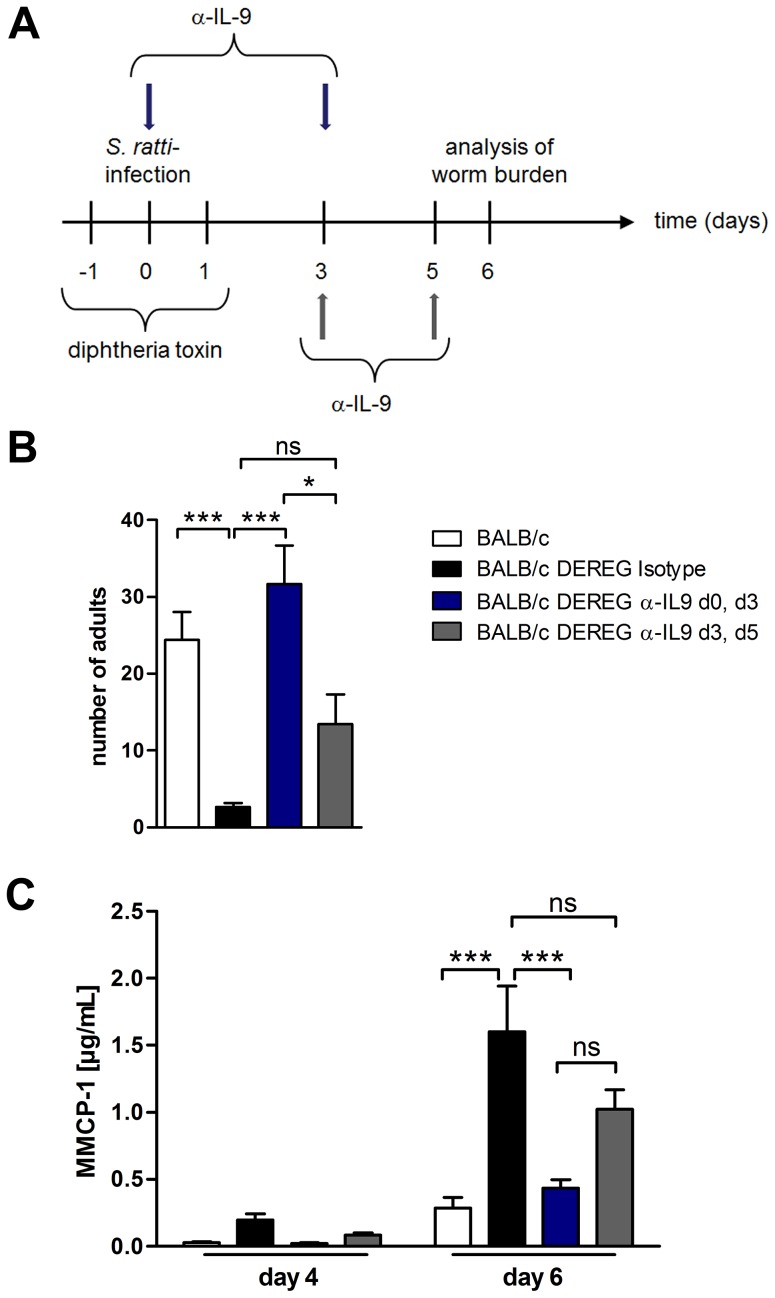
Depletion of IL-9 during *S. ratti* infection in BALB/c DEREG mice at different time points. **A:** Experimental setup for α-IL-9 treatment either early (blue arrows) or late (grey arrows) in *S. ratti* infection is shown. **B:** Graph shows number of parasitic adults in the small intestine of BALB/c (white), Treg-depleted, isotype treated BALB/c DEREG (black), Treg-depleted early α-IL-9 treated BALB/c DEREG (dark blue) and Treg-depleted late α-IL-9 treated BALB/c DEREG (dark grey) mice on day 6 p.i. Shown are the combined results of three independent experiments (n = 14). **C:** Concentrations of MMCP-1 in the serum of infected mice on day 4 and day 6 p.i. are shown as combined results of two independent experiments (n = 9). Asterisks indicate significant difference of the mean analyzed by one-way ANOVA with Bonferroni post test (* p≤0.05, *** p≤0.001).

To further elucidate the kinetics of IL-9 production relevant for the early mast cell degranulation and protection we neutralized IL-9 during *S. ratti* infection in Treg-depleted BALB/c mice at different time points (Figure 8A). IL-9 neutralization throughout infection, achieved by anti-IL-9 injections at the day of infection and at day 3 p.i., abrogated the beneficial effect of Treg depletion in BALB/c mice as expected (Figure 8B). Neutralization of endogenous IL-9 in Treg-depleted BALB/c mice at later time points i.e. starting day 3 p.i. with a second injection at day 5 p.i. led to an intermediate phenotype. The numbers of parasitic *S. ratti* adults in Treg-depleted mice receiving late IL-9 neutralization were significantly lower than parasite burden in Treg-depleted BALB/c mice where IL-9 was neutralized from the beginning of infection. We still observed a trend towards higher parasite burden compared to the low parasite number achieved upon Treg depletion in the presence of endogenous IL-9, suggesting that IL-9 was important also after day 3 p.i. Kinetics of mast cell degranulation reciprocally reflected the parasite burden (Figure 8C). Treg depletion increased mast cell degranulation and the additional early IL-9 neutralization but not the delayed IL-9 neutralization prevented that rapid mast cell degranulation.

To identify the source of IL-9, we performed intracellular cytokine staining (Figure S5). In line with a recent report [42] IL-9 was produced by a very low frequency of CD4^+^ and CD4^−^ cells in the spleen and mesenteric lymph node of nematode- mice and Treg depletion increased the frequency of both, CD4^+^ and CD4^−^ IL-9-expressing cells. Taken together, these results show that increased production of IL-9 during the first days of infection was central for establishment of enhanced resistance in Treg-depleted BALB/c DEREG mice.

### Foxp3^+^ Treg control IL-9-mediated mast cell activation in *S. ratti*-infected BALB/c mice

To provide a causative link between the observed IL-9 dependent mast cell degranulation (Figures 6–8) and the improved resistance in Treg-depleted BALB/c DEREG mice, we crossed BALB/c DEREG mice to a recently generated mast cell-deficient mouse strain [61]. In Cpa3^CRE^ knockin mice, Cre-recombinase is expressed under control of the carboxypeptidase A3 (Cpa3) promoter which results in constitutive and complete deletion of mucosal and connective tissue mast cells, and mild reduction in splenic basophil numbers, whereas other features of the immune system are normal. This is in contrast to Kit-mutant mouse models that carry significant additional immune aberrations due to the dysfunctional Kit [41], [62]. Crossing heterozygous BALB/c DEREG to heterozygous BALB/c Cpa3^CRE^ mice yielded four different genotypes in the first generation offspring: BALB/c Cpa3^WT^ that contained normal mast cell numbers and did not express DTR on Treg, BALB/c DEREG Cpa3^WT^ that contained normal mast cell numbers but expressed DTR on Treg, BALB/c Cpa3^CRE^ that were mast cell-deficient but did not express DTR on Treg, and BALB/c DEREG Cpa3^CRE^ that were mast cell-deficient and expressed DTR on Treg. All groups were treated with DT and subsequently infected with *S. ratti*.

**Figure 9:**
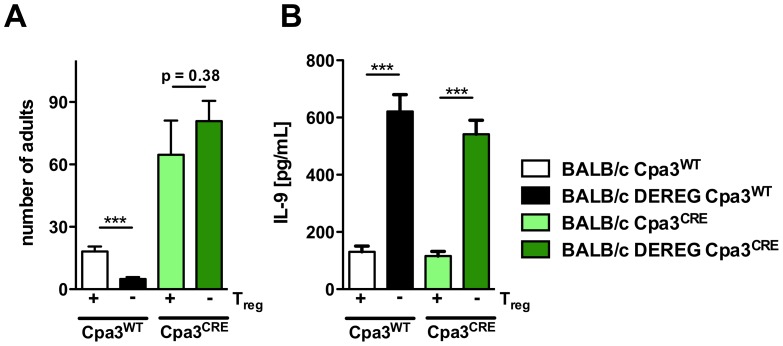
Role of mast cells during *S. ratti i*nfection in Treg-depleted BALB/c mice. **AB:** BALB/c Cpa3^WT^ (white bars), BALB/c DEREG Cpa3^WT^ (black bars), BALB/c Cpa3^CRE^ (light green bars) and BALB/c DEREG Cpa3^CRE^ (dark green bars) mice were treated with DT on three consecutive days starting one day before s.c infection with 2000 *S. ratti* iL3. **A:** Number of parasitic adults in the small intestine was counted on day 6 p.i. Depicted are the combined results of three independent experiments (n≥12) and error bars show SEM. **B:** Splenocytes were prepared at day 6 p.i. and cultured in the presence of α-CD3 for 72 h. IL-9 concentration in the supernatant was quantified by ELISA. Unstimulated splenocytes did not secrete detectable IL-9. Shown are the combined results of three independent experiments (n≥12). **AB:** Asterisks indicate significant difference of the mean analyzed by students *t* test (*** p≤0.001).

Comparison of parasite burden in the presence of Treg in mast cell-competent BALB/c Cpa3^WT^ mice and mast cell-deficient BALB/c Cpa3^CRE^ mice demonstrated that mast cell-deficiency as such increased parasite burden (Figure 9A). While these data are in agreement with older studies that employed Kit-mutant mice as models for mast cell deficiency in *S. ratti* [38] and *S. venezuelensis* [48] infection, it was identified a key role for mast cells in protection against intestinal nematode infection, given that several suggested roles for mast cells have not been confirmed in Kit-independent mast cell deficient mice [61], [63], [64], [65].

Treg depletion in the presence of mast cells reduced parasite burden day at 6 p.i. (Figure 9A) and increased production of IL-9 (Figure 9B) as we had shown before (Figures 2, 4, 7, 8). Additional mast cell deficiency completely abrogated improved resistance in Treg-depleted BALB/c DEREG Cpa3^CRE^ mice (Figure 9A), while IL-9 production was still increased. Thus increased IL-9 production that was caused by Treg depletion in vivo was only translated into improved resistance in the presence of mast cells. These results strongly suggest that IL-9 activated mast cells represent the central effector cells mediating rapid expulsion of *S. ratti* in Treg-depleted BALB/c mice.

## Discussion

Comparing the role of Foxp3^+^ Treg during nematode-induced immune evasion in fully susceptible BALB/c and C57BL/6 mice we report a pronounced difference. Parasite survival in BALB/c mice was strictly dependent on the presence of Foxp3^+^ Treg whereas in C57BL/6 mice prolonged parasite survival was still established in the absence of Foxp3^+^ Treg. Different impact of Foxp3^+^ Treg depletion on parasite burden did not reflect different kinetics of parasite eradication. Analysis of *S. ratti* larvae in the feces showed that the kinetics of infection and clearance in the presence of Treg were comparable in both strains. While *S. ratti* larval output was reduced upon Treg depletion in BALB/c mice throughout infection, larval output remained unchanged in Treg-depleted C57BL/6 mice until natural clearance of infection.

Different impact of Foxp3^+^ Treg on host defense was not due to differences in Treg induction or depletion nor to DT-mediated side effects. Expansion of Foxp3^+^ Treg was comparable in both strains displaying maximal numbers at day 2 p.i. in the lymph nodes draining the site of infection and at day 14 p.i. in the lymph nodes draining the intestine i.e. the environment of parasitic adults. Also contraction of the Foxp3^+^ Treg compartment to naïve levels after resolution of infection was alike. Thus spatial and temporal induction of Foxp3^+^ Treg correlated with parasite migration through the host in BALB/c and C57BL/6 mice. Depletion efficacy and repopulation kinetics were also identical in BALB/c and C57BL/6 mice, as we controlled by monitoring Foxp3^+^CD4^+^ Treg in peripheral blood and in the lymphatic organs. Expanding Treg displayed an activated CD103^+^ phenotype [44] in both strains. Neither BALB/c nor C57BL/6 mice showed preferential expansion of natural, thymus-derived Treg since frequencies of Helios^+^ and Neuropilin^+^ Treg remained stable during infection. Biologic function of Foxp3^+^ Treg in vivo was apparent in both strains because Treg depletion led to a distinctive increase in the cellular and humoral type 2 immune responses: i.e. production of IL-4, IL-13, IL-3, IL-10, IgM and IgE. Although a functional type 2 immune response has been reported to be central for the control of *Strongyloides* infection [51], [52], these effector molecules obviously did not mediate accelerated eradication of *S. ratti* upon Treg depletion in C57BL/6 mice.

One concern upon comparing BALB/c and C57BL/6 mice is that the latter are more susceptible to *S. ratti* infection, displaying between two and five times more parasitic adults in the small intestine as BALB/c mice infected with the same iL3 batch. Immune control of this high parasite burden may be difficult even in the context of improved immune responses. To address this possibility, we compared the effect of Treg depletion in low dose infected C57BL/6 mice to BALB/c mice receiving the standard infection dose. C57BL/6 mice that carried 10 parasites per mouse still did not respond to Treg depletion with improved parasite expulsion whereas BALB/c mice that carried two times more parasites displayed drastically reduced parasite numbers upon Treg depletion. Thereby, we ruled out the higher parasite burden as such as explanation for the missing effect of Treg depletion in C57BL/6 mice. Furthermore, we show that in principle reduction of a high parasite burden can be achieved because IL-9 treatment reduced the naturally high parasite burden in C57BL/6 mice and depletion of granulocytes increased the naturally lower parasite burden in BALB/c mice more than two-fold but still Treg depletion led to improved *S. ratti* expulsion.

We identified IL-9-driven rapid degranulation of mucosal mast cells as the underlying mechanism causing improved resistance in Treg-depleted BALB/c mice because of the following evidence:

- IL-9 and mast cell degranulation were significantly up-regulated in Treg-depleted BALB/c mice that displayed reduced parasite burden but not in Treg-depleted C57BL/6 mice that displayed unchanged parasite burden.
- Neutralization of IL-9 but not neutralization of IL-13 or depletion of granulocytes reverted the parasite burden to high levels and delayed the mast cell degranulation to normal kinetics in Treg-depleted BALB/c mice.
- Treg depletion caused increased IL-9 production in mast cell-competent and mast cell-deficient mice to the same extent. However, the increased IL-9 production was translated into reduced parasite burden only in the presence of mast cells.

These results strongly suggest that within our model of murine *S. ratti* infection Treg controlled mast cell degranulation via control of IL-9 that acted on mast cells and not via cellular interaction, as was shown in a model of IgE induced anaphylaxis [66]. We did not observe differential regulation of any other effector relevant for infection control apart from IL-9 production and mast cell activation. Therefore, we argue that these mechanisms are causative for the observed parasite reduction, yet additional mediators involved in the chain of reactions that lead to parasite clearance cannot be excluded.

Regarding the potential cellular source(s) of this protective IL-9, Treg [67], [68], Th2, Th9 [55], [69] and innate lymphoid cells (ILC) [70] have been described. We observed both IL-9 producing CD4^+^ and CD4^−^ cells during infection at very low frequencies. While IL-9 production by Treg themselves is unlikely in our system as we observed increased IL-9 production specifically upon Treg depletion, our results suggest contribution to IL-9 production by Th9 cells and CD4^−^ ILC. In strong support of this reasoning, a novel IL-9 reporter mouse revealed IL-9 production at comparably low frequencies by Th9 cells and ILC during *N. brasiliensis* infection [42].

By neutralizing IL-9 at different time points post infection we further show that IL-9 neutralization in Treg-depleted BALB/c mice later than day 2 p.i. did not restore the susceptible phenotype of BALB/c mice containing normal Treg frequencies. This finding suggests that the IL-9 that was relevant for rapid mast cell degranulation and parasite expulsion was produced during the first days of infection. As Treg depletion also reduced worm burden only if Treg were depleted during the first days of infection [14], we suggest that Treg control early IL-9 production in BALB/c mice.

Our results consent with the established function for IL-9 in the recruitment of mast cells and promotion of mastocytosis in general [56], [71]. The particular importance of IL-9 and IL-9-mediated mast cell activation for host defense during gastrointestinal nematode infection, however, depends on the nematode species and the genetic background of the host. *N. brasiliensis*-infected IL-9-deficient 129×C57BL/6 (F_2_) mice displayed reduced MMCP-1 activity in the small intestine but still expelled the parasites as efficiently as wildtype mice in one study [56]. A recently generated IL-9-deficient mouse model on BALB/c background displayed reduced mast cell numbers but was additionally more susceptible to *N. brasiliensis* infection [42]. Moreover, adoptive transfer of Th9 cells reduced *N. brasiliensis* parasite burden in either IL-9 deficient or T cell- and B cell- deficient RAG^−/−^ mice, thus demonstrating a fundamental role of T cell-derived IL-9 in eradication of intestinal nematode infection.

This is in line with earlier studies showing the general importance of IL-9 by any source in control of intestinal nematode infection. Forced increase in systemic IL-9 concentration by injection of a IL-9 transgenic (tg) cell line accelerated expulsion of *Trichuris muris* in C57BL/6 mice [57] and IL-9 tg FVB mice displayed faster clearance of *T. muris* infection in the context of increased mastocytosis [57]. Reciprocal neutralization of endogenous IL-9 that was achieved by vaccination against IL-9 before nematode infection rendered resistant C57BL/6 mice susceptible for *T. muris* infection [59]. IL-9 tg mice also expulsed *Trichinella spiralis* rapidly from the small intestine in a mast cell dependent manner [58]. Increased *T. spiralis* expulsion in IL-9 treated C57BL/6 mice was correlated to increased muscle contractility and MMCP-1 production in the small intestine [60].

We analyzed the impact of IL-9 during *Strongyloides* infection and showed that administration of IL-9 reduced parasite burden while neutralization of IL-9 reciprocally increased parasite burden in the small intestine of *S. ratti*-infected BALB/c and C57BL/6 mice. Increased parasite burden upon IL-9 neutralization was accompanied by reduced mast cell degranulation in both strains, suggesting that endogenous IL-9 triggered mast cell degranulation during *S. ratti* infection in BALB/c and C57BL/6 mice in the presence of normal Treg frequencies. Employing Kit-independent mast cell-deficient mice, we provided direct evidence that the increase in IL-9 production observed in Treg-depleted BALB/c mice triggered mast cells to mediate rapid expulsion of *S. ratti* subsequently. We hypothesize that the non-significant increase in IL-9 production observed in C57BL/6 mice upon Treg depletion was not sufficient to increase mast cell degranulation and enhance parasite expulsion. Since treatment with recombinant IL-9 readily reduced parasite burden also in C57BL/6 mice, the function of IL-9 during *S. ratti* infection apparently is comparable in both strains whereas the control of IL-9 production in vivo is maintained differently.

The different impact of in vivo Treg depletion on IL-9 production was not Treg-intrinsic. Purified BALB/c- and C57BL/6-derived Treg suppressed IL-2 but also IL-9 secretion by syngenic and allogenic effector cells to the same extent. Although an older study reported that BALB/c-derived CD25^+^CD4^+^ Treg suppressed in vitro proliferation of syngenic effector T cells more efficiently than BALB/c-derived Treg, this difference was tracked down to C57BL/6-derived effector T cells that were more difficult to suppress by both, BALB/c and C57BL/6 Treg [72].

Since we detected no Treg-intrinsic differences in vitro, the differential regulation of IL-9 in BALB/c and C57BL/6 mice that was reproducibly apparent in vivo can be explained either by a limited capacity of C57BL/6 mice to produce IL-9 or by the presence of redundant regulatory elements in C57BL/6 mice that continued to control IL-9 production in the absence of Foxp3^+^ Treg. Along the lines of this working hypothesis it is tempting to suggest that the increased susceptibility of C57BL/6 mice to *S. ratti* infection in general was due to reduced availability of IL-9 as a consequence of more stringent regulation. The reduced susceptibility to *S. ratti* infection displayed by BALB/c mice on the other hand may reflect increased availability of IL-9 due to the less stringent regulation of endogenous IL-9 production and subsequent mast cell degranulation.

Genetically determined differences in the impact of Treg on immune regulation during infection as well as strain-specific differences in the impact of IL-9-driven and mast cell-mediated intestinal inflammation have been reported in other models. Comparing expansion and phenotype of Foxp3^+^ Treg and Foxp3^−^ effector T cells during *Litomosoides sigmodontis* infection in susceptible BALB/c and resistant C57BL/6 mice Taylor et al. described comparable recruitment and expansion of Foxp3^+^ Treg in both strains [13]. Nevertheless, *in vivo* effector T cell proliferation and expression of the costimulatory molecule GITR was more pronounced in semi-permissive C57BL/6 mice. Since depletion of Treg in susceptible BALB/c mice prior to infection reduced the parasite burden at day 60 p.i., it is conceivable that Treg promoted nematode-induced immune evasion in BALB/c mice and contributed to the susceptibility of this genotype. Also the impact of Treg depletion or Treg transfer on the course of *Toxoplasma gondii* [73] and *Mycobacterium tuberculosis* [74] infection was more pronounced in BALB/c than in C57BL/6 mice, again suggesting different levels of Treg redundancy in different mouse strains.

IL-9 induced mastocytosis and subsequent increase in intestinal permeability mediates intestinal anaphylaxis in a murine model for food allergy independent of other Th2 associated effectors [75]. This intestinal anaphylaxis can be induced by vaccination with Alum emulsified Ovalbumin prior intra-gastric Ovalbumin challenge selectively in BALB/c mice and not in C57BL/6 mice [76]. It remains to be elucidated if the resistance to IL-9-driven and mast cell-induced intestinal anaphylaxis in C57BL/6 mice reflected a different function of IL-9 and mast cells in the different mouse strains or different control of IL-9 production.

We did not identify a putative regulatory element that maintained control of IL-9 production, mast cell degranulation and thus *S. ratti* survival in C57BL/6 mice in the absence of Foxp3^+^ Treg so far. The thorough analysis of the different modes of immune regulation induced during nematode infection in inbred mouse models may eventually improve the interpretation of the diverse results observed in studies within the helminth-infected human population.

## Materials and Methods

### Ethics statement

Animal experimentation was conducted at the animal facility of the Bernhard Nocht Institute for Tropical Medicine in agreement with the German animal protection law under the supervision of a veterinarian. The experimental protocols have been reviewed and approved by the responsible federal health Authorities of the State of Hamburg, Germany, the “Behörde für Gesundheit und Verbraucherschutz” permission number 54/10 and 55/13. Mice were sacrificed by cervical dislocation under deep CO_2_ narcosis. DEREG mice were generated by injecting a BAC directly into fertilized C57BL/6 oocytes [26]. The resulting C57BL/6 DEREG founder was backcrossed to BALB/c thus ensuring similar insertion sites of the BAC into the genome in both strains. Cpa3^CRE^ mice were generated by homologous recombination of Cre recombinase into the Cpa3 locus and backcrossed to BALB/c mice for 19 generations [61]. Heterozygous C57BL/6 DEREG mice were mated with wildtype C57BL/6 mice, and heterozygous BALB/c DEREG mice were bred with wildtype BALB/c or intercrossed with heterozygous BALB/c Cpa3^CRE^ mice in the animal facilities of the Bernhard Nocht Institute for Tropical Medicine in order to provide littermates for the experiments. Wistar rats were purchased from Charles River (Sulzfeld, Germany). Animals were kept in individually ventilated cages under specific pathogen-free conditions and used at the age of 6–10 wk (mice) or 4–8 wk (rats).

### *S. ratti* life cycle and infections

The *S. ratti* cycle was maintained by serial passage of *S. ratti* through Wistar rats. *S. ratti* iL3 were purified from charcoal feces cultures as described [29]. Prior to infection, iL3 were stored overnight in PBS supplemented with penicillin (100 U/mL) and streptomycin (100 µg/mL). BALB/c DEREG, C57BL/6 DEREG and non-transgenic BALB/c and C57BL/6 littermates were infected by s.c. injection of 2000 or 200 purified iL3 in 30 µl PBS into the hind footpad. Groups of mice received 0.5 µg DT (Merck, Darmstadt, Germany) dissolved in PBS (pH 7.4) i.p. on three consecutive days, starting one day prior to *S. ratti* infection. Treg depletion was routinely controlled by analysis of peripheral blood samples for GFP, Foxp3, and CD4 expression at day 2 p.i. (and day 6 p.i. for the experiment shown in Figure 3). Recombinant IL-9 (eBioscience, San Diego, USA) was administered i.p. to BALB/c and C57BL/6 (200 ng/mouse and time point) at the indicated time points. For neutralization of IL-9 BALB/c and C57BL/6 mice or BALB/c DEREG and littermate control mice received 100 µg anti–IL-9 mAb (clone MM9C1, BioXCell, West Lebanon, USA) i.p. at the indicated time points. For depletion of granulocytes mice received 300 µg anti-Gr1 (clone RB6-8C5) i.p. either one day before infection or at day 3 p.i.

### Analysis of parasite burden

To count the number of adult parasitic females in the gut, the small intestine was flushed slowly with tap water to remove feces, sliced open longitudinally and incubated at 37°C for 3 h in a petri dish with tap water. The released adult females were collected by centrifugation for 5 min at 1200 rpm and counted. To quantify the release of *S. ratti* larvae by infected mice, the feces of individual mice was collected over 24 h periods and DNA from representative 200 mg samples was extracted as described [77]. 200 ng DNA was used as a template for qPCR specific for *S. ratti* 28 S ribosomal RNA gene as described [33].

### Cytokine production

For analysis of cellular responses mice were sacrificed at the indicated time points and spleen and MLN were dissected. A total of 2×10^5^ spleen or MLN cells were cultured in 3–5 replicates 96-well round-bottom plates in RPMI 1640 medium supplemented with 10% FCS, 20 mM HEPES, L-glutamine (2 mM), and gentamicin (50 µg/mL) at 37°C and 5% CO_2_. The cells were stimulated for 72 h with medium, anti-mouse CD3 (145-2C11, 1 µg/mL), or *S. ratti* iL3 lysate (20 µg/mL) that was prepared as described [33]. The supernatant was harvested for analysis of cytokine production by ELISA. The supernatants of spleen cell cultures derived from infected mice incubated with medium did not contain detectable concentrations of IL-3, IL-4, IL-9, IL-10 or IL-13.

### ELISA

For analysis of serum antibodies and mouse mast cell protease-I (MMCP-I), blood was collected from infected mice at the indicated time points and allowed to coagulate for 1 h at room temperature (RT). Serum was collected after centrifugation at 10,000× *g* for 10 min at RT and stored at −20°C for further analysis. *Strongyloides*-specific Ig in the serum was quantified by ELISA, as described [33]. Briefly, 50 µL/well *S. ratti* Ag lysate (2.5 µg/mL) in PBS was coated overnight at 4°C on Microlon ELISA plates (Greiner, Frickenhausen, Germany). Plates were washed four times with PBS 0.05% Tween 20 and blocked by incubation with PBS 1% BSA for 2 h at RT. Serial dilutions of sera in PBS 0.1% BSA were incubated in duplicate, adding 50 µL/well overnight at 4°C. Plates were washed five times, and *Strongyloides*-specific Ig was detected by incubation with 50 µL/well horseradish peroxidase (HRP)-conjugated anti-mouse IgM (Zymed Karlsruhe, Germany) for 1 h at RT. Plates were washed five times and developed by incubation with 100 µL/well tetramethylbenzidine 0.1 mg/ml, 0.003% H_2_O_2_ in 100 mM NaH_2_PO_4_ (pH 5.5) for 2.5 min. Reaction was stopped by addition of 25 µL/well 2 M H_2_SO_4_, and OD at 450 nm (OD_450_) was measured. The titer was defined as the highest dilution of serum that led to an OD_450_ above the doubled background. Background was always below 0.15 OD_450_. Concentration of IgE was quantified using the IgE ELISA kit (BD, Heidelberg Germany) and MMCP-I in serum was detected using the MMCP-I ELISA Ready-SET-Go kit (eBioscience, San Diego, USA) both according to the manufacturers recommendations. IL-3, IL-4, IL-10 and IL-13) in culture supernatants were measured using DuoSet ELISA development kits (R&D Systems, Wiesbaden, Germany), according to the manufacturer's instructions. IL-9 detection was performed by coating with 2 µg/mL anti-IL-9 Ab (BD, Heidelberg, Germany) overnight at 4°C. Plates were blocked with 10% FCS/0.05%Tween/PBS for 2 h at RT. Samples and recombinant IL-9 standard (Peprotech, Hamburg, Germany) were incubated overnight and detection was performed with an anti-IL-9-biotin AB (BD, Heidelberg, Germany) for 1 h at RT and subsequent Streptavidin-HRP incubation for 20 min before development with 100 µL/well tetramethylbenzidine 0.1 mg/ml, 0.003% H_2_O_2_ in 100 mM NaH_2_PO_4_ (pH 5.5). The reaction was stopped after 10 min by adding 25 µL of 2 M H_2_SO_4_.

### Flow cytometry

To prevent unspecific binding of mAbs, all samples were pre incubated with 25 µL of Fc block at 4°C for 10 min. Surface staining was carried out for 20 min at 4°C using Allophycocyanin (APC)-labeled anti-CD4 (clone RM4-5; BD Heidelberg, Germany) or Brilliant Violet 510-labeled anti-CD4 (clone RM4-5, Biolegend), Phycoerythrin (PE)-labeled anti-CD 304 (Neuropilin-1, clone 3E12, Biolegend), PE- or FITC-labeled anti-CD103 (clone 2E7, Biolegend), Peridinin chlorophyll protein-cyanine5-labeled anti-CD11b (clone M1/70, BD, Heidelberg, Germany.) and PE-labeled anti-Gr-1 (clone 1A8, BD). For intracellular staining cells were permeabilized with 250 µl fixation/permeabilization buffer for 30 min at 4°C, washed with permeabilization buffer and stained with PE- or Alexa Fluor 700-labeled anti-Foxp3 (clone FJK-16s, eBiosciences, SanDiego, USA) and APC–labeled anti-Helios (clone 22F6, Biolegend). For analysis of Neuropilin and Helios expression cells were also stained for 30 min on ice with LIVE/DEAD Fixable Blue Dead Cell Stain Kit for UV excitation according to the manufacturers recommendation (Life Technologies, Darmstadt, Germany). Samples were measured on a BD FACSCalibur or LSRII and analyzed using FlowJo software.

### Statistical analysis

Statistical analysis was performed with GraphPad Prism software (San Diego) using the One way ANOVA followed by the Bonferroni post test or the students *t* test to calculate the significance of differences between multiple or between two groups, respectively. The data are presented as mean ± SEM; *p*≤0.05 was considered statistically significant.

## Supporting Information

Figure S1 **Gating strategy for** **Figure 1** **.** Naïve or *S. ratti*-infected mice were sacrificed and single cell lymph node cultures prepared. **AB:** Cells were stained with Allophycocyanin (APC)-labeled anti-CD4 (clone RM4-5) and Phycoerythrin (PE)-labeled isotype control or PE-labeled anti-CD103 (clone 2E7) and analyzed on a BD FACSCalibur. **A:** Shown are representative dotplots for identification of Treg and Teff as CD4^+^GFP^+^ and CD4^+^GFP^−^ cells in the lymphocyte gate. **B:** Shown are representative dotplots for identification of activated Treg as CD4^+^GFP^+^CD103^+^ cells in the lymphocyte gate. **C:** Lymph node cells were stained with LIVE/DEAD Fixable Blue Dead Cell Stain, followed by surface staining with PE-labeled anti CD304 (Neuropilin-1; clone 3E12) and Brilliant Violet 510-labeled anti-CD4 (clone RM4-5). After fixation and permeabilization, cells were stained with Alexa Fluor 700-labeled anti-Foxp3 (clone FJK-16s) and APC-labeled anti-Helios (clone 22F6). Samples were measured on a LSRII. Shown are representative dotplots for exclusion of doublets and dead cells and subsequent identification of Helios^+^ and Neuropilin^+^ cells within the CD4^+^ Foxp3^+^population.
(PDF)

Figure S2 **Unchanged resistance to S.** ***ratti*** **infection in Treg-depleted C57BL/6 DEREG mice during low dose infections.** BALB/c (white bars), BALB/c DEREG (black bars), C57BL/6 (light grey bars), and C57BL/6 DEREG (dark grey bars) mice were treated with DT and infected s.c. with either 2000 *S. ratti* iL3 (BALB/c) or 200 iL3 (C57BL/6). Numbers of parasitic adults in the small intestine were counted on day 6 p.i. Shown are the combined results of two independent experiments (n = 4 for BALB/c and BALB/c DEREG; n = 9 for C57BL/6 and C57BL/6 DEREG). Numbers show significant differences of the mean analyzed by students *t* test.
(PDF)

Figure S3 **Improved resistance in Treg-depleted BALB/c DEREG mice upon granulocyte depletion after the tissue migrating phase.** BALB/c (white bars) and BALB/c DEREG (black bars) mice were treated with DT and received an injection of anti-Gr1 mAb (300 µg/mouse) at day 3 of *S. ratti* infection (**A**). Numbers of adult parasitic females in the small intestine were counted on day 6 p.i. (**B**). Shown are the combined results of two independent experiments (n = 4–8). Asterisks indicate significant difference of the mean analyzed by students *t* test (** p≤0.01).
(PDF)

Figure S4 **In vitro suppression by BALB/c- and C57BL/6-derived Treg.** CD4^+^CD25^+^ Treg were purified from BALB/c (open bars) and C57BL/6 (black bars) spleens by magnetic cell sorting according to the manufacturers recommendations (Miltenyi Biotec, Bergisch Gladbach, Germany). Purity was between 86% and 98%. 1×10^5^ splenocytes derived from day 6 *S. ratti*-infected and Treg-depleted BALB/c DEREG (left panel) or C57BL/6 DEREG (right panel) mice were cultured in the presence of anti-mouse CD3 (145-2C11, 1 µg/mL) and Treg in indicated cellular ratio in 96-well round-bottom plates in RPMI 1640 medium supplemented with 10% FCS, 20 mM HEPES, L-glutamine (2 mM), and gentamicin (50 µg/mL) at 37°C and 5% CO_2_ for 72 h in 3–5 replicates. Supernatant was harvested and IL-2 and IL-9 were quantified by ELISA. To compare several experiments maximal cytokine production in one given experiment was set to 100% and percent suppression was calculated subsequently. Cytokine production by anti-CD3 activated BALB/c DEREG splenocytes was in the range of 185 pg/mL to 525 pg/mL (IL-2) and 58 pg/ml to 400 pg/mL (IL-9); anti-CD3 activated C57BL/6 splenocytes produced 90 pg/mL to 164 pg/mL (IL-2) and 46 pg/mL to 77 pg/mL (IL-9). Unstimulated splenocytes did not produce detectable IL-2 and IL-9. Shown are the combined results of 4–8 individual experiments, error bar shows SEM between individual experiments (n = 8 for IL-2 by BALB/c- and C57BL/6-derived effectors; n = 7 for IL-9 by BALB/c-derived effectors; n = 4 for IL-9 by C57BL/6-derived effectors).
(PDF)

Figure S5 **Intracellular IL-9 staining.** **S5A:** Representative gating strategy for exclusion of doublets and dead cells is shown. **S5B:** Representative plots for identification of IL-9^+^ CD4^+^ cells in Th9 polarized spleen cell cultures. **S5C:** Representative plots for identification of IL-9^+^ CD4^+^ and CD4^−^ cells in spleen and MLN from *S. ratti* infected and DT treated BALB/c and BALB/c DEREG mice. **Th9 polarization:** For Th9 polarization T cells were isolated from spleens using CD4^+^ T cell Isolation Kit II (MACS Miltenyi Biotec) according to the manufacturers recommendation. 1×10^7^ CD4^+^ T cells were cultured in 10 mL RPMI medium on purified anti-mouse CD3ε (clone 145-2C11) coated cell culture dishes in the presence of 5 µg/mL purified anti-mouse CD28 (clone 37.51), 10 µg/mL purified anti-mouse IFN-γ (clone AN-18), 20 ng/mL recIL-2, 20 ng/mL recIL-4, 10 ng/mL human TGF-β (all Biolegend) for 3 days at 37°C and 5% CO_2_. **Detection of** ***S. ratti*** **-induced IL-9 producing cells:** To detect IL-9 producing cells ex vivo BALB/c and BALB/c DEREG mice were infected s.c. with 2000 iL3 *S. ratti*. Mice received 0,5 µg DT on three consecutive days starting one day before infection. Mice were sacrificed day 6 p.i and reduced parasite burden in the small intestine of Treg depleted BALB/c DEREG mice compared to non depleted BALB/c mice was verified (data not shown). Spleens and mesenteric lymph nodes (MLN) were prepared, stimulated and analyzed for IL-9 production. **Intracellular IL-9 staining:** 2–3×10^6^ cells were incubated for 6 h with 500 ng/mL PMA (Phorbol-12-myristat-13-acetat, Sigma) and 500 ng/mL Ionomycin (Sigma) in the presence of 1× Brefeldin A Solution (Biolegend) at 37°C and 5% CO_2_. Cells were first stained with LIVE/DEAD Fixable Blue Dead Cell Stain (1∶1000, Invitrogen) in PBS for 30 min at 4°C. Cells were washed and subsequently surface stained with PE-Cy7-labeled anti-CD4 (clone RM4-5, Biolegend) for 30 min at 4°C. Cells were fixed and permeabilized using Foxp3-Fixation/Permeabilization Kit (eBioscience) according to the manufacturers recommendation. Intracellular IL-9 was detected using Alexa Fluor 647-labeled anti-mouse IL-9 (clone RM9A4, Biolegend) for 30 min at room temperature. Cells were washed and analyzed on a LSRII.
(PDF)
