# Supplementary material for: Foxp3+ Regulatory T Cells Delay Expulsion of Intestinal Nematodes by Suppression of IL-9-Driven Mast Cell Activation in BALB/c but Not in C57BL/6 Mice
Source: PLoS Pathog. 2014 Feb 6;10(2):e1003913. doi: 10.1371/journal.ppat.1003913 (PMC3916398; doi:10.1371/journal.ppat.1003913)
Supplement: Figure S1 — Gating strategy for Figure 1 . Naïve or S. ratti-infected mice were sacrificed and single cell lymph node cultures prepared. AB: Cells were stained with Allophycocyanin (APC)-labeled anti-CD4 (clone RM4-5) and Phycoerythrin (PE)-labeled isotype control or PE-labeled anti-CD103 (clone 2E7) and analyzed on a BD FACSCalibur. A: Shown are representative dotplots for identification of Treg and Teff as CD4+GFP+ and CD4+GFP− cells in the lymphocyte gate. B: Shown are representative dotplots for identification of activated Treg as CD4+GFP+CD103+ cells in the lymphocyte gate. C: Lymph node cells were stained with LIVE/DEAD Fixable Blue Dead Cell Stain, followed by surface staining with PE-labeled anti CD304 (Neuropilin-1; clone 3E12) and Brilliant Violet 510-labeled anti-CD4 (clone RM4-5). After fixation and permeabilization, cells were stained with Alexa Fluor 700-labeled anti-Foxp3 (clone FJK-16s) and APC-labeled anti-Helios (clone 22F6). Samples were measured on a LSRII. Shown are representative dotplots for exclusion of doublets and dead cells and subsequent identification of Helios+ and Neuropilin+ cells within the CD4+ Foxp3+population. (PDF) [file ppat.1003913.s001.pdf]

Figure S1

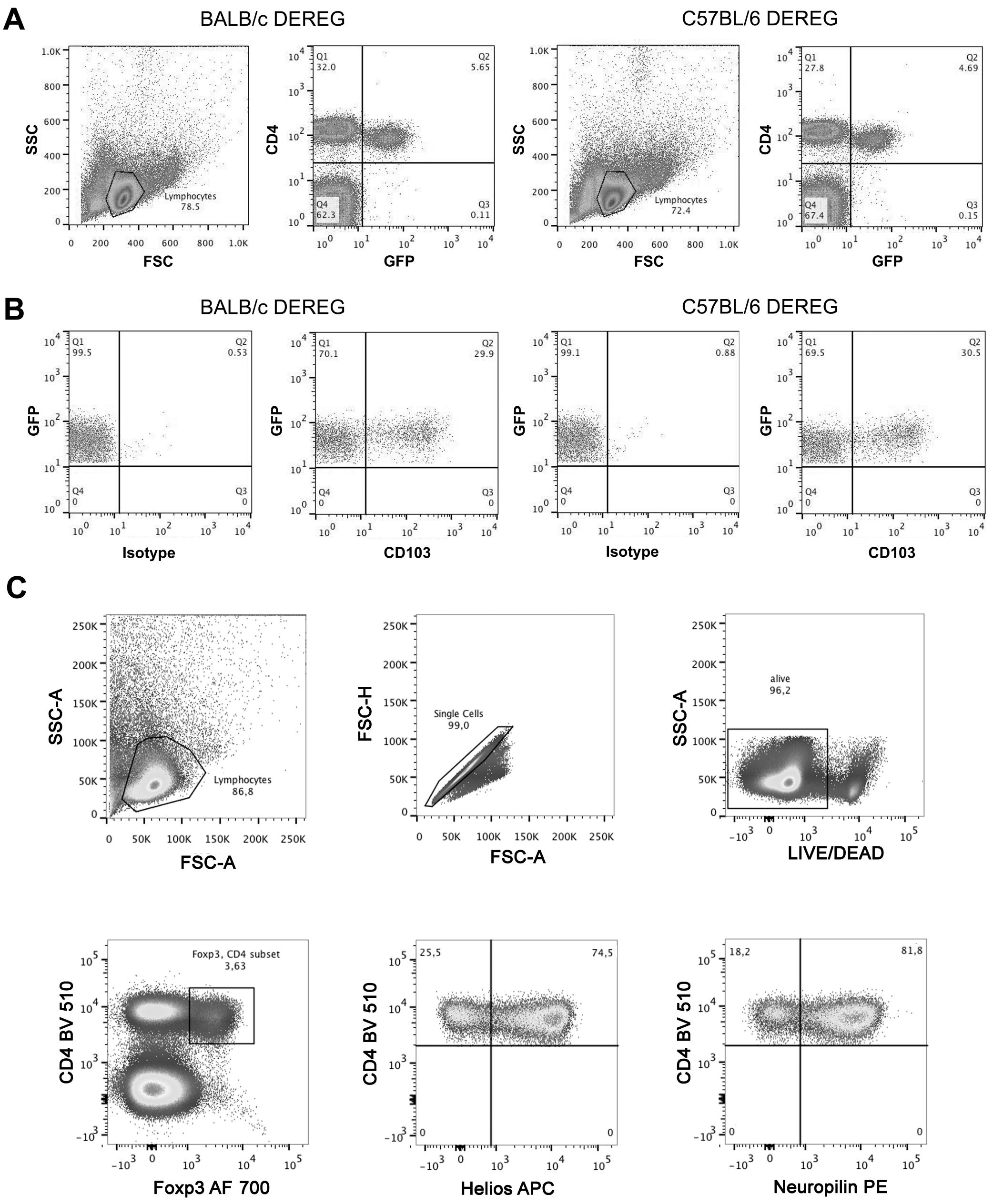

S1: Gating strategy for Figure 1

Naïve or *S. ratti*-infected mice were sacrificed and single cell lymph node cultures prepared. **AB**: Cells were stained with Allophycocyanin (APC)-labeled anti-CD4 (clone RM4-5) and Phycoerythrin (PE)-labeled isotype control or PE-labeled anti-CD103 (clone 2E7) and analyzed on a BD FACSCalibur. **A:** Shown are representative dotplots for identification of Treg and Teff as $CD4^+GFP^+$ and $CD4^+GFP^-$ cells in the lymphocyte gate. **B:** Shown are representative dotplots for identification of activated Treg as $CD4^+GFP^+CD103^+$ cells in the lymphocyte gate. **C:** Lymph node cells were stained with LIVE/DEAD Fixable Blue Dead Cell Stain, followed by surface staining with PE-labeled anti CD304 (Neuropilin-1; clone 3E12) and Brilliant Violet 510-labeled anti-CD4 (clone RM4-5). After fixation and permeabilization, cells were stained with Alexa Fluor 700-labeled anti-Foxp3 (clone FJK-16s) and APC-labeled anti-Helios (clone 22F6). Samples were measured on a LSRII. Shown are representative dotplots for exclusion of doublets and dead cells and subsequent identification of $Helios^+$ and $Neuropilin^+$ cells within the $CD4^+$ $Foxp3^+$population.
