# Supplementary material for: Foxp3+ Regulatory T Cells Delay Expulsion of Intestinal Nematodes by Suppression of IL-9-Driven Mast Cell Activation in BALB/c but Not in C57BL/6 Mice
Source: PLoS Pathog. 2014 Feb 6;10(2):e1003913. doi: 10.1371/journal.ppat.1003913 (PMC3916398; doi:10.1371/journal.ppat.1003913)
Supplement: Figure S2 — Unchanged resistance to S. ratti infection in Treg-depleted C57BL/6 DEREG mice during low dose infections. BALB/c (white bars), BALB/c DEREG (black bars), C57BL/6 (light grey bars), and C57BL/6 DEREG (dark grey bars) mice were treated with DT and infected s.c. with either 2000 S. ratti iL3 (BALB/c) or 200 iL3 (C57BL/6). Numbers of parasitic adults in the small intestine were counted on day 6 p.i. Shown are the combined results of two independent experiments (n = 4 for BALB/c and BALB/c DEREG; n = 9 for C57BL/6 and C57BL/6 DEREG). Numbers show significant differences of the mean analyzed by students t test. (PDF) [file ppat.1003913.s002.pdf]

Figure S2

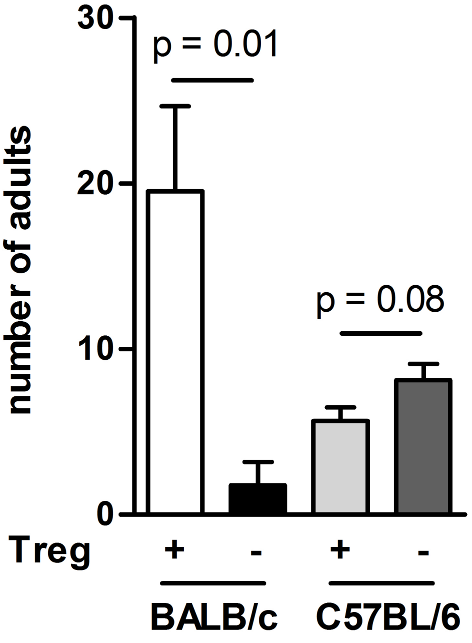

S2: Unchanged resistance to S. *ratti* infection in Treg depleted C57BL/6 DEREG mice during low dose infections

BALB/c (white bars), BALB/c DEREG (black bars), C57BL/6 (light grey bars), and C57BL/6 DEREG (dark grey bars) mice were treated with DT and infected s.c. with either 2000 *S. ratti* iL3 (BALB/c) or 200 iL3 (C57BL/6). Number of parasitic adults in the small intestine was counted on day 6 p.i. Shown are the combined results of two independent experiments (n = 4 for BALB/c and BALB/c DEREG; n = 9 for C57BL/6 and C57BL/6 DEREG). Numbers show significant difference of the mean analyzed by students *t* test.
