# Supplementary material for: Foxp3+ Regulatory T Cells Delay Expulsion of Intestinal Nematodes by Suppression of IL-9-Driven Mast Cell Activation in BALB/c but Not in C57BL/6 Mice
Source: PLoS Pathog. 2014 Feb 6;10(2):e1003913. doi: 10.1371/journal.ppat.1003913 (PMC3916398; doi:10.1371/journal.ppat.1003913)
Supplement: Figure S3 — Improved resistance in Treg-depleted BALB/c DEREG mice upon granulocyte depletion after the tissue migrating phase. BALB/c (white bars) and BALB/c DEREG (black bars) mice were treated with DT and received an injection of anti-Gr1 mAb (300 µg/mouse) at day 3 of S. ratti infection (A). Numbers of adult parasitic females in the small intestine were counted on day 6 p.i. (B). Shown are the combined results of two independent experiments (n = 4–8). Asterisks indicate significant difference of the mean analyzed by students t test (** p≤0.01). (PDF) [file ppat.1003913.s003.pdf]

Figure S3

A:

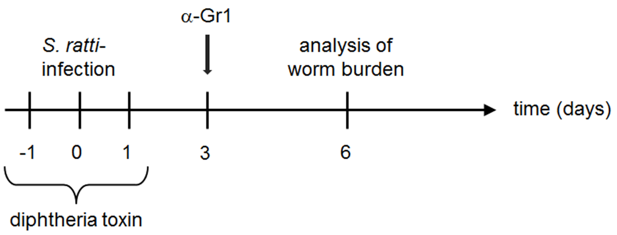

B:

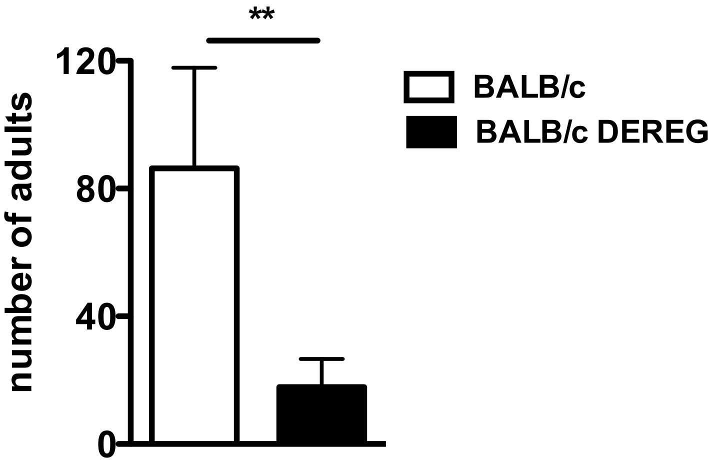

S3: Improved resistance in Treg depleted BALB/c DEREG mice upon granulocyte depletion after the tissue migrating phase

BALB/c (white bars) and BALB/c DEREG (black bars) mice were treated with DT and received an injection of anti-Gr1 mAb (300 µg/mouse) at day 3 of *S. ratti* infection **(A).** Number of adult parasitic females in the small intestine was counted on day 6 p.i. **(B)**. Shown are the combined results of two independent experiments (n = 4-8). Asterisks indicate significant difference of the mean analyzed by students *t* test (** $p \leq 0.01$).
