# Supplementary material for: Foxp3+ Regulatory T Cells Delay Expulsion of Intestinal Nematodes by Suppression of IL-9-Driven Mast Cell Activation in BALB/c but Not in C57BL/6 Mice
Source: PLoS Pathog. 2014 Feb 6;10(2):e1003913. doi: 10.1371/journal.ppat.1003913 (PMC3916398; doi:10.1371/journal.ppat.1003913)
Supplement: Figure S4 — In vitro suppression by BALB/c- and C57BL/6-derived Treg. CD4+CD25+ Treg were purified from BALB/c (open bars) and C57BL/6 (black bars) spleens by magnetic cell sorting according to the manufacturers recommendations (Miltenyi Biotec, Bergisch Gladbach, Germany). Purity was between 86% and 98%. 1×10^5^ splenocytes derived from day 6 S. ratti-infected and Treg-depleted BALB/c DEREG (left panel) or C57BL/6 DEREG (right panel) mice were cultured in the presence of anti-mouse CD3 (145-2C11, 1 µg/mL) and Treg in indicated cellular ratio in 96-well round-bottom plates in RPMI 1640 medium supplemented with 10% FCS, 20 mM HEPES, L-glutamine (2 mM), and gentamicin (50 µg/mL) at 37°C and 5% CO2 for 72 h in 3–5 replicates. Supernatant was harvested and IL-2 and IL-9 were quantified by ELISA. To compare several experiments maximal cytokine production in one given experiment was set to 100% and percent suppression was calculated subsequently. Cytokine production by anti-CD3 activated BALB/c DEREG splenocytes was in the range of 185 pg/mL to 525 pg/mL (IL-2) and 58 pg/ml to 400 pg/mL (IL-9); anti-CD3 activated C57BL/6 splenocytes produced 90 pg/mL to 164 pg/mL (IL-2) and 46 pg/mL to 77 pg/mL (IL-9). Unstimulated splenocytes did not produce detectable IL-2 and IL-9. Shown are the combined results of 4–8 individual experiments, error bar shows SEM between individual experiments (n = 8 for IL-2 by BALB/c- and C57BL/6-derived effectors; n = 7 for IL-9 by BALB/c-derived effectors; n = 4 for IL-9 by C57BL/6-derived effectors). (PDF) [file ppat.1003913.s004.pdf]

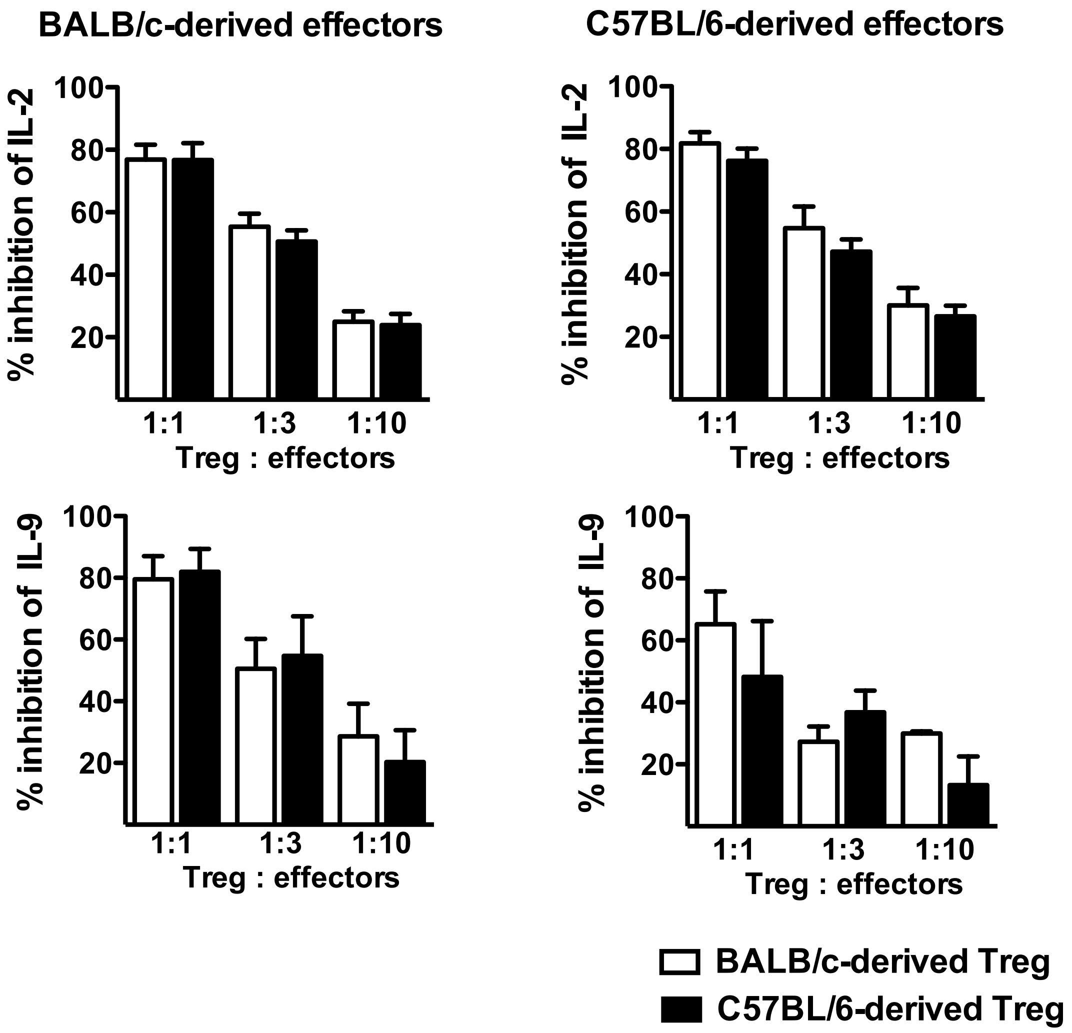

Figure S4: In vitro suppression by BALB/c- and C57BL/6-derived Treg
$CD4^+CD25^+$ Treg were purified from BALB/c (open bars) and C57BL/6 (black bars) spleens by magnetic cell sorting according to the manufacturers recommendations (Miltenyi Biotec, Bergisch Gladbach, Germany). Purity was between 86% and 98%. 1 x 10^5 splenocytes derived from day 6 *S. ratti*-infected and Treg-depleted BALB/c DEREG (left panel) or C57BL/6 DEREG (right panel) mice were cultured in the presence of anti-mouse CD3 (145-2C11, 1 µg/mL) and Treg in indicated cellular ratio in 96-well round-bottom plates in RPMI 1640 medium supplemented with 10% FCS, 20 mM HEPES, L-glutamine (2 mM), and gentamicin (50 µg/mL) at 37°C and 5% CO_2 for 72h in 3-5 replicates. Supernatant was harvested and IL-2 and IL-9 were quantified by ELISA. To compare several experiments maximal cytokine production in one given experiment was set to 100% and percent suppression was calculated subsequently. Cytokine production by anti-CD3 activated BALB/c DEREG splenocytes was in the range of 185 pg/mL to 525 pg/mL (IL-2) and 58 pg/ml to 400 pg/mL (IL-9); anti-CD3 activated C57BL/6 splenocytes produced 90 pg/mL to 164 pg/mL (IL-2) and 46 pg/mL to 77 pg/mL (IL-9). Unstimulated splenocytes did not produce detectable IL-2 and IL-9. Shown are the combined results of 4-8 individual experiments, error bar shows SEM between individual experiments (n = 8 for IL-2 by BALB/c- and C57BL/6-derived effectors; n = 7 for IL-9 by BALB/c-derived effectors; n = 4 for IL-9 by C57BL/6-derived effectors).
