# Supplementary material for: Foxp3+ Regulatory T Cells Delay Expulsion of Intestinal Nematodes by Suppression of IL-9-Driven Mast Cell Activation in BALB/c but Not in C57BL/6 Mice
Source: PLoS Pathog. 2014 Feb 6;10(2):e1003913. doi: 10.1371/journal.ppat.1003913 (PMC3916398; doi:10.1371/journal.ppat.1003913)
Supplement: Figure S5 — Intracellular IL-9 staining. S5A: Representative gating strategy for exclusion of doublets and dead cells is shown. S5B: Representative plots for identification of IL-9+ CD4+ cells in Th9 polarized spleen cell cultures. S5C: Representative plots for identification of IL-9+ CD4+ and CD4− cells in spleen and MLN from S. ratti infected and DT treated BALB/c and BALB/c DEREG mice. Th9 polarization: For Th9 polarization T cells were isolated from spleens using CD4+ T cell Isolation Kit II (MACS Miltenyi Biotec) according to the manufacturers recommendation. 1×10^7^ CD4+ T cells were cultured in 10 mL RPMI medium on purified anti-mouse CD3ε (clone 145-2C11) coated cell culture dishes in the presence of 5 µg/mL purified anti-mouse CD28 (clone 37.51), 10 µg/mL purified anti-mouse IFN-γ (clone AN-18), 20 ng/mL recIL-2, 20 ng/mL recIL-4, 10 ng/mL human TGF-β (all Biolegend) for 3 days at 37°C and 5% CO2. Detection of S. ratti -induced IL-9 producing cells: To detect IL-9 producing cells ex vivo BALB/c and BALB/c DEREG mice were infected s.c. with 2000 iL3 S. ratti. Mice received 0,5 µg DT on three consecutive days starting one day before infection. Mice were sacrificed day 6 p.i and reduced parasite burden in the small intestine of Treg depleted BALB/c DEREG mice compared to non depleted BALB/c mice was verified (data not shown). Spleens and mesenteric lymph nodes (MLN) were prepared, stimulated and analyzed for IL-9 production. Intracellular IL-9 staining: 2–3×10^6^ cells were incubated for 6 h with 500 ng/mL PMA (Phorbol-12-myristat-13-acetat, Sigma) and 500 ng/mL Ionomycin (Sigma) in the presence of 1× Brefeldin A Solution (Biolegend) at 37°C and 5% CO2. Cells were first stained with LIVE/DEAD Fixable Blue Dead Cell Stain (1∶1000, Invitrogen) in PBS for 30 min at 4°C. Cells were washed and subsequently surface stained with PE-Cy7-labeled anti-CD4 (clone RM4-5, Biolegend) for 30 min at 4°C. Cells were fixed and permeabilized using Foxp3-Fixation/Permeabilization Kit (eBioscienc [file ppat.1003913.s005.pdf]

Figure S5: Intracellular IL-9 staining

S5A: Representative gating strategy for exclusion of doublets and dead cells is shown.
S5B: Representative plots for identification of IL-9$^+$ CD4$^+$ cells in Th9 polarized spleen cell cultures.
S5C: Representative plots for identification of IL-9$^+$ CD4$^+$ and CD4$^-$ cells in spleen and MLN from *S. ratti* infected and DT treated BALB/c and BALB/c DEREG mice.

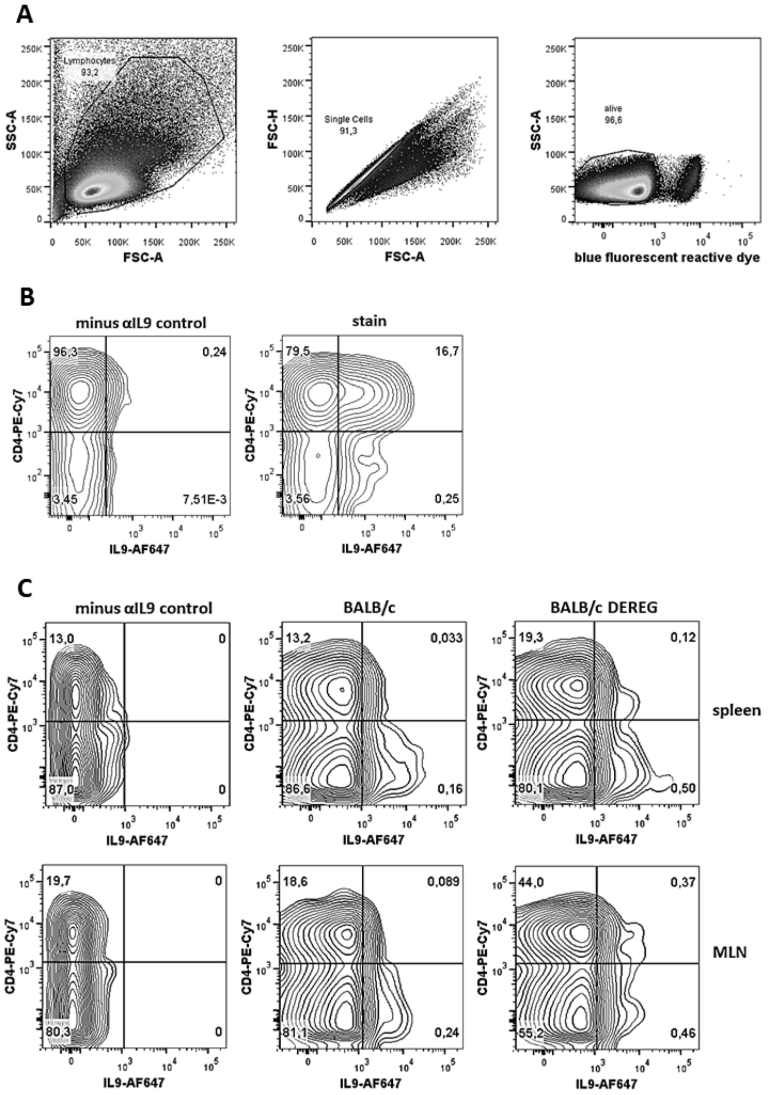

Th9 polarization:

For Th9 polarization T cells were isolated from spleens using CD4$^+$ T cell Isolation Kit II (MACS Miltenyi Biotec) according to the manufacturers recommendation. 1x10^7 CD4$^+$ T cells were cultured in 10 mL RPMI medium on purified anti-mouse CD3ε (clone 145-2C11) coated cell culture dishes in the presence of 5 µg/mL purified anti-mouse CD28 (clone 37.51), 10 µg/mL purified anti-mouse IFN-γ (clone AN-18), 20 ng/mL recIL-2, 20 ng/mL recIL-4, 10 ng/mL human TGF-β (all Biolegend) for 3 days at 37°C and 5% CO_2.

Detection of *S. ratti*-induced IL-9 producing cells:

To detect IL-9 producing cells ex vivo BALB/c and BALB/c DEREG mice were infected s.c. with 2000 iL3 *S. ratti*. Mice received 0,5 µg DT on three consecutive days starting one day before infection. Mice were sacrificed day 6 p.i and reduced parasite burden in the small intestine of Treg depleted BALB/c DEREG mice compared to non depleted BALB/c mice was verified (data not shown). Spleens and mesenteric lymph nodes (MLN) were prepared, stimulated and analyzed for IL-9 production.

Intracellular IL-9 staining:

2-3x10^6 cells were incubated for 6 h with 500 ng/mL PMA (Phorbol-12-myristat-13-acetat, Sigma) and 500 ng/mL Ionomycin (Sigma) in the presence of 1x Brefeldin A Solution (Biolegend) at 37°C and 5% CO_2. Cells were first stained with LIVE/DEAD Fixable Blue Dead Cell Stain (1:1000, Invitrogen) in PBS for 30 min at 4°C. Cells were washed and subsequently surface stained with PE-Cy7-labeled anti-CD4 (clone RM4-5, Biolegend) for 30 min at 4°C. Cells were fixed and permeabilized using Foxp3-Fixation/Permeabilization Kit (eBioscience) according to the manufacturers recommendation. Intracellular IL-9 was detected using Alexa Fluor 647-labeled anti-mouse IL-9 (clone RM9A4, Biolegend) for 30 min at room temperature. Cells were washed and analyzed on a LSRII.
